# Water Drops Sliding Over Arrays of Janus Micropillars With Hydrophilic Tops: A New Mechanism of Drop Charging

**DOI:** 10.1002/smll.202511728

**Published:** 2026-03-11

**Authors:** Fahimeh Darvish, Mark Isaacs, Sajjad Shumaly, Lea Delance, Hans‐Jürgen Butt

**Affiliations:** ^1^ Max Planck Institute for Polymer Research (MPI‐P) Mainz Germany; ^2^ Institute for Condensed Matter Physics Technische Universität Darmstadt Darmstadt Germany; ^3^ HarwellXPS, Research Complex at Harwell Rutherford Appleton Lab Didcot United Kingdom; ^4^ Department of Chemistry University College London London United Kingdom

**Keywords:** drop charge deposition, Janus micropillar, photolithography, satellite droplets, APTES and PFOTS vapor deposition, RIE plasma–surface interactions, XPS imaging

## Abstract

When water drops slide over a flat hydrophobic solid surface, negative charges are spontaneously deposited at the rear of the drop, and the drops gain a positive charge. This phenomenon is known as slide electrification. Here, we demonstrate an alternative mechanism of spontaneous charge separation in sliding drops. Therefore, arrays of Janus micropillars were fabricated by photolithography. Each micropillar has a hydrophilic top created by reactive ion etching (RIE) and aminopropyltriethoxysilane (APTES) deposition, while the walls and substrate were hydrophobized by 1H,1H,2H,2H‐perfluorooctyltrichlorosilane (PFOTS). Drops on these arrays show high apparent contact angles, remain in the Cassie state, and still contact the hydrophilic top faces. Sliding drops acquire charge, with polarity and magnitude governed by top‐surface chemistry, including NH_3_^+^/NH_4_^+^ formation and PFOTS removal after the RIE plasma exposure, and dense NH_2_ groups formation from thermally activated APTES vapor deposition. Unlike flat surfaces, high‐speed reflection microscopy reveals tiny satellite droplets left on each pillar that evaporate within one second. Consequently, charge separation occurs within the liquid phase between the primary drop and deposited satellites, rather than directly at the contact line.

## Graphical abstract

A water drop slides over a distance of a few centimeters across arrays whose top surfaces exhibit a contact angle below 10°. The arrays are fabricated with hydrophilic tops and hydrophobic sidewalls and substrate, forming Janus structures. As the drop moves, it generates a spontaneous electric charge and leaves behind tiny satellite droplets that evaporate rapidly.

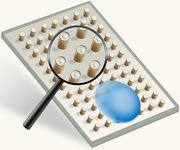

## 1 Introduction

Water drops sliding on an inclined hydrophobic surface naturally acquire electrical charges, while depositing countercharges on the surface. This phenomenon is called *slide* or *contact electrification*. As a result of charging, drop potentials can reach 1 kV or more, depending on the thickness of the substrate [1, 2]. Slide electrification causes drop friction and alters drop motion [3]. It is considered for generating electric energy [4, 5] and is relevant for inkjet printing [6], self‐driving microfluidic [3, 7], and sensing [8].

Many experiments indicate that the deposited charges originate from the surface charges spontaneously formed at the solid‐water interface. When a solid surface comes into contact with aqueous electrolyte, an electrical double layer is formed. The electrical double layer consists of a layer of bound surface charges and a diffuse layer of countercharges shielding the surface charges, the thickness of which is characterized by the Debye length. At a receding contact line, the electric double layer separates. Some of the surface charges are deposited on the dewetted surface and the countercharges remain in the drop [9, 10, 11, 12].

Usually, the bound surface charges are formed by dissociation of surface groups or by adsorption of ions. For hydrophobic surfaces, it is still debated how surface charges are formed [13, 14, 15, 16, 17]. Zeta potential measurements [14], potentiometric titration [18, 19] and surface force experiments [20] demonstrate that they are negatively charged [21].

Slide electrification has previously been observed primarily on hydrophobic materials [5, 9, 22] or superhydrophobic surfaces [7, 23, 24] but not on hydrophilic surfaces. It is not clear why slide electrification has been primarily observed on hydrophobic surfaces. One reason could be due to practical rather than fundamental reasons, namely the low apparent contact angle and surface conductivity of hydrophilic surfaces. The observation itself is surprising, because hydrophilic surfaces are usually highly charged. An explanation has been proposed by Ratschow et al. [25]. Based on an earlier theory [26] they found that for receding contact angles greater than 90° the local charge density near the contact line increases assuming constant surface potential. Vice versa, for contact angles below 90° the local charge density decreases. Assuming that some of the charges remain on the free surface, this would result in fewer deposited charges. A second possible effect is that deposited charges may be neutralized more quickly due to the conductivity provided by a continuous adsorbed water layer on hydrophilic surfaces. Adsorbed water can change the surface conductivity by orders of magnitude [27, 28, 29]. On hydrophobic surfaces the water layer is absent or reduced. A practical reason why slide electrification has not been detected on hydrophilic surfaces is that at a low apparent contact angle, drops are wide, easily disintegrate when sliding [30, 31] and their motion and charge are difficult to measure.

**FIGURE 1:**
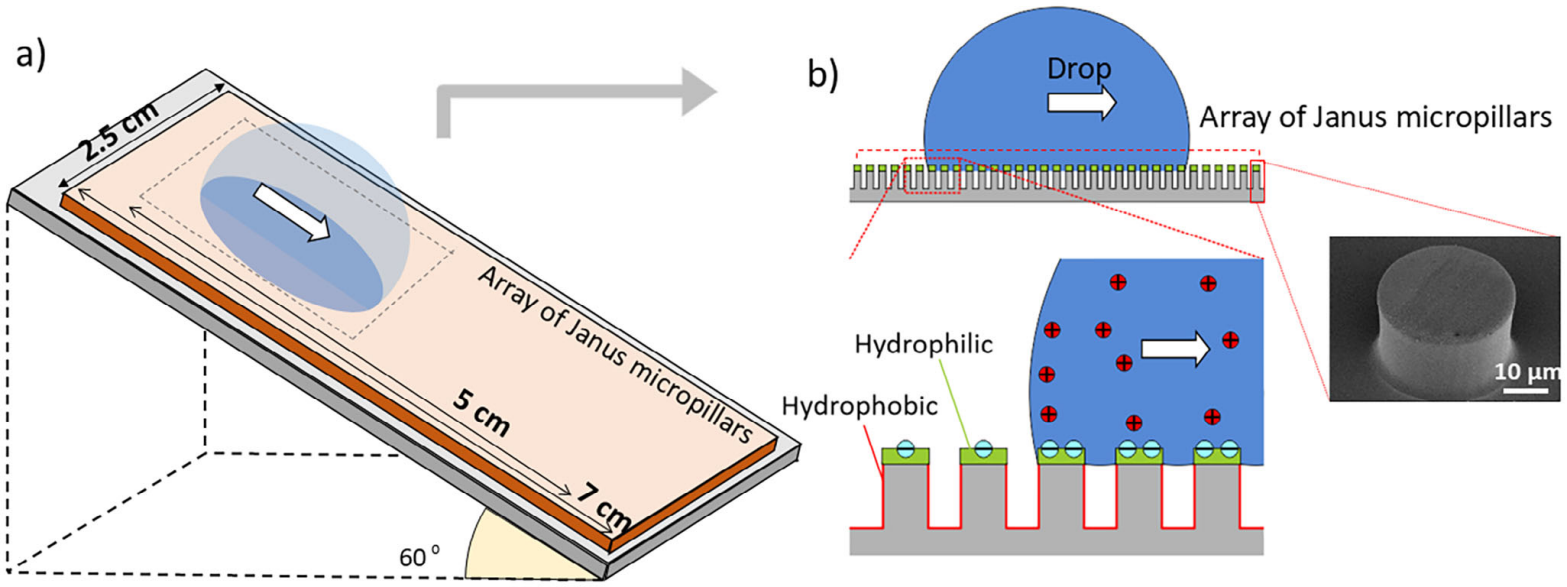
Schematic of a sliding water drop on an array of Janus micropillars mounted on a tilted plate. a) On a homogeneous, flat, hydrophilic surface, the drop would spread; in contrast, it forms a high apparent contact angle on a surface patterned with Janus micropillars. b) Zoomed view of the Janus micropillar arrays showing the wetting contrast and drop‐charging mechanism, with an SEM image of a single micropillar (10 µm scale bar).

In order to overcome these problems and to study slide electrification on hydrophilic surfaces we fabricated arrays of Janus micropillars with hydrophobic side walls and a hydrophobic substrate but hydrophilic top faces (Figure 1). On such arrays the drop is only in contact with the top faces. An air layer is formed over the hydrophobic regions, and the drop remains in the Cassie state. As a result, it has a high apparent contact angle. Surface‐conductivity‐driven discharge via a continuous adsorbed water film is eliminated because the hydrophilic top faces are spatially separated by hydrophobic (PFOTS) regions and a Cassie air layer, which hinders the formation of a percolating water film across the surface.

Different types of arrays of Janus micropillar had been described before, but none have addressed sliding drops on hydrophilic surfaces [32, 33, 34, 35, 36, 37, 38, 39, 40] (Table S1). For our purpose, a large area of 7 × 2.5 cm^2^ was required to allow the drop to slide. To achieve this, we had to develop a new method of preparing the arrays. The array may also be important for other applications than drop charging. Their dual wettability (vertically or laterally) makes them interesting in microfluidics [39], cell migration [36] and adhesion [41], bio‐medicine [42] through micro and nano patterning methods like soft lithography [43], E‐beam writing, and laser printing [44, 45].

We hydrophilized the top of Janus arrays using two methods: RIE plasma functionalization and APTES deposition. In both cases, the side walls and substrate were hydrophobized by PFOTS. Using plasma RIE/DeepRIE is a necessary step in micropatterning, but it is worth noting that this RIE process can lead to the absorption of positive charges through the plasma‐surface interaction^55^. This effect is indirectly detected using the sliding drop charging measurement.

Water drops placed on Janus micropillar arrays remained in the Cassie state, meaning drops contacted only the top faces and not the substrate. As a result, the apparent contact angle observed on a length scale larger than the microstructure was >90°. The method is promising for other types of micropatterning, such as nanoparticle deposition on micropillar arrays [46], as it allows precise control of chemistry, roughness, adhesion, and wettability exclusively on the tops of arrays, while keeping the sidewall chemistry independently adjustable.

Here, we did indeed observe spontaneous drop charging on arrays of micropillars with hydrophilic top surfaces. But instead of answering the original question, we found an entirely new charging mechanism. Satellite droplets are left behind on the top of each micropillar. These droplets contain an excess of negative or positive charge, depending on the surface chemistry of the micropillar. The actual charge separation process does not occur at the receding contact line but when the tiny satellite droplets separate from the primary drop. The primary drop accumulates the opposite charge.

## 2 Result and Discussion

### 2.1 Water Contact Angle

The advancing and receding contact angles on PFOTS‐SU8 flat surfaces were Θ*_a_* = 115° and Θ*_r_* = 95°, respectively (Table 1). On PFOTS‐SU8 arrays, the apparent advancing and receding contact angles increased to 146° and 127°, respectively. Drops were in the Cassie state and no water touched the substrate.

**TABLE 1:** Apparent advancing and receding contact angles of water on the three types of micropillar arrays, Θ*_a_^app^* and Θ*_r_^app^*, and the corresponding contact angles on the respective flat SU8 surfaces, Θ*_a_* and Θ*_r_*. The schematic of all samples is shown above the table.

Sample name	Flat surface		Arrays	
Contact angle	**Θ*_a_***	**Θ*_r_***	**Θ*_a_^app^***	**Θ*_r_^app^***
PFOTS‐on‐SU8 (PFOTS‐SU8)	115 ± 1°	95 ± 2°	146 ± 4°	127 ± 4°
			**Janus arrays**	
N_2_ Plasma‐on‐PFOTS‐SU8 (N_2_‐SU8)	<10°	<10°	141±1°	124±3°
APTES‐on‐ N_2_ Plasma‐on‐PFOTS‐SU8 (APTES‐SU8)	<10°	<10°	139° ± 4°	122 ± 3°

Upon exposure to plasma or deposition of APTES, the PFOTS‐SU8 flat surface became hydrophilic, exhibiting an advancing contact angle of 10° or less immediately after modification. RIE plasma removed the PFOTS layers even at the weakest operating parameters (40 W, 5 s) and samples became hydrophilic. Arrays of Janus micropillars produced by the plasma and APTES had average contact angles of 140° and 123°. The drop was in a Cassie state, despite the top face being hydrophilic with contact angles Θ*_a_* and Θ*_r_* below 10°. To make the Janus arrays superhydrophobic, PFOTS coating on the sidewalls and substrate was essential since bare glass and SU8 were hydrophilic [47]. Similarly, when the entire surface of PFOTS‐SU8 arrays was treated with N_2_ RIE plasma or APTES, they became fully hydrophilic, preventing the movement of water drops.

On flat SU8 deposited with APTES without plasma activation of SU8, we measured Θ_a_ = 63° and Θ_r_ = 34°. However, that surface became hydrophilic (contact angles < 10°) when the PFOTS‐SU8 surface was activated by the N_2_ RIE plasma before APTES deposition (the contact angleswere measured immediately after sample preparation).

### 2.2 Drop Charging

**FIGURE 2:**
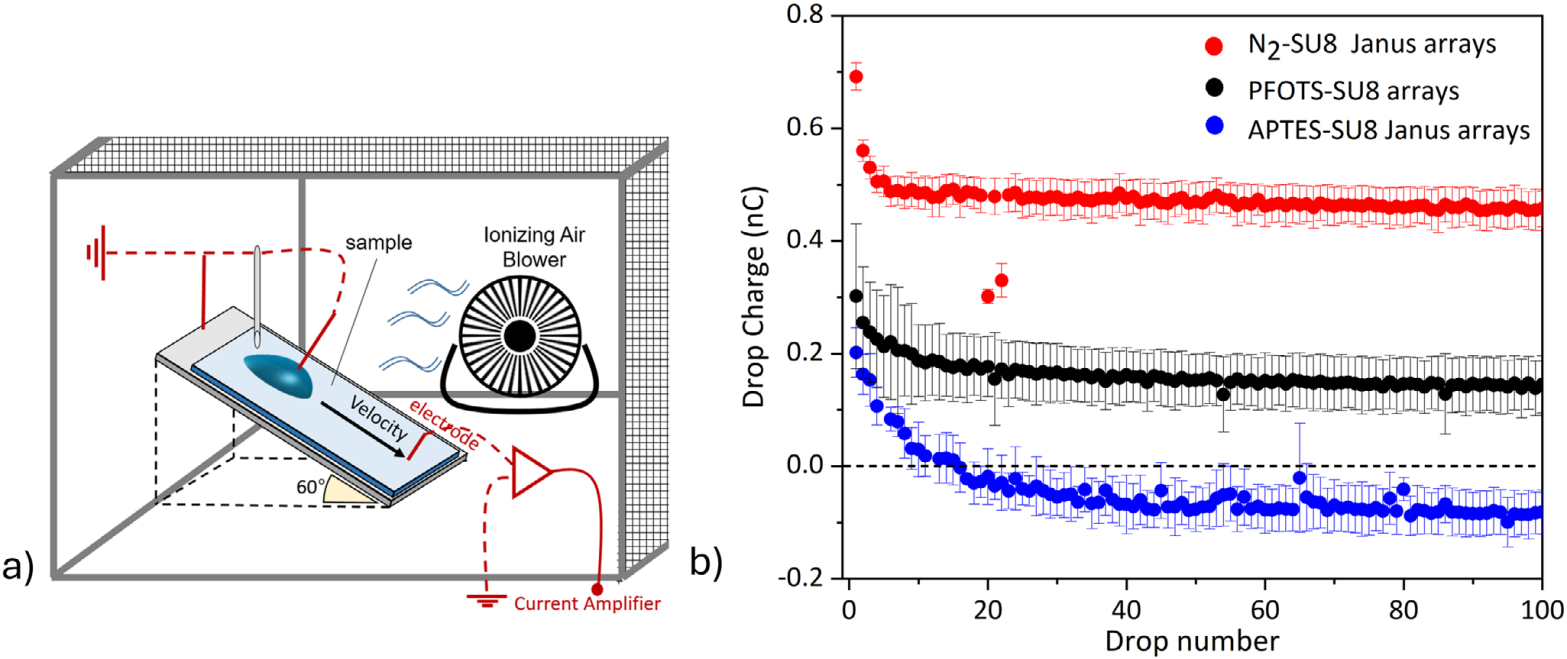
Measuring the charge of sliding water drops. a) Schematic of the setup to measure the charge of sliding water drops. The drop volume was 45±1 µL, the interval between drops was 4 ± 0.2 s, and drops slid over 5 cm‐long samples. Sample tilt angle was 60°. b) Charge versus drop number measured on PFOTS‐SU8 arrays (black data points), N_2_‐SU8 Janus arrays (red data points) and APTES‐SU8 Janus arrays (blue data points). Each point corresponds to the net charge of a drop measured after sliding 5 cm. The drop number therefore indexes repeated, nominally identical sliding events on the same track.

Drop charges were measured for a series of water drops using a home‐made experimental setup (Figure 2a) for the three types of samples (Table 1). The drop charge (Q) as a function of drop number for all surfaces was highest for the first drop and decreased monotonically before saturating after ten or few tens of drops (Figure 2b). For each drop, we record the current pulse generated when the drop contacts the second electrode. The drop charge is obtained by time‐integrating this pulse. Thus, each data point in Figure 2b corresponds to one individual drop in the sequence.

On PFOTS‐SU8 arrays the first water drop had a positive charge of ≈0.3 nC. After the ≈20^th^ drop, the charge saturated at ≈0.18 nC. Drop charges saturate with the number of drops because the second and subsequent drops hit an already charged surface. As a result, they are charged negatively on their advancing side. Since the drop charge is the sum of the charge deposited at the back and the charge collected at the front [3], the drop charge saturates. With enough time between successive drops, the surface discharges and becomes neutral. Once neutralized, the next drop acquires a similar charge to the first drop. The recovery time depends on the substrate.

On flat PFOTS‐SU8 surfaces, drop charge‐vs‐number plots showed a higher charge for the first drop (1.1 nC) but rather similar steady state drop charges (Figure S1a). In prior studies, the lower drop charges observed in the Cassie state have been explained by a reduced real effective liquid‐solid contact area [48, 49]. In the Cassie state, the real contact area is only a fraction of the apparent contact area. The drop charge increases with the area that the rear contact line passes over while receding and is therefore also proportional to the real contact area. To examine this effect, the length and width of the apparent contact area were calculated from side‐view video analysis of sliding drops [50, 51, 52]. As shown in Figure S2, the apparent drop width on PFOTS‐SU8 arrays and on flat PFOTS‐SU8 surfaces was almost identical. On average, the real contact area over which the rear contact line passes is only φ = 0.2 of the apparent contact area. Jiang et al. pointed out that the receding contact line of a drop on a structured (Cassie‐state) surface can be comparable to that observed on flat surfaces [53, 54]. Therefore, we attribute the change in drop charge to differences in the real contact area.

On Janus arrays produced by N_2_‐RIE plasma, we measured even higher drop charges (saturating around ≈0.48, red points in Figure 2b) than on the flat PFOTS‐SU8 surface (which saturated at ≈0.3, Figure S1), despite having a pillar structure with a solid fraction of φ = 0.2. Thus, charge separation is also more pronounced on hydrophilic surfaces. Steady state was achieved after just five drops, approximately four times faster than on PFOTS‐SU8 arrays.

One possible explanation for this higher charging and faster approach to steady state is N_2_ RIE plasma treatment. During interaction of SU8 with the N_2_ RIE plasma, positive ions are implanted near the surface by the RIE plasma [55, 56, 57, 58, 59]. Such a positive charge is confirmed through zeta potential and XPS measurements (section 2.4.2). This observation agrees with our previous study, in which an increasing drop charge was detected on PFOTS deposited on a glass that had been exposed to a low‐pressure plasma cleaner (40 kHz) and placed close to the electrode [59]. RIE plasma reactors are designed so that samples must be placed on the power electrode to maximize ion bombardment [55]. Due to the higher mobility of electrons compared to ions, electrons escape the plasma more easily, leading to the formation of a positive space‐charge region near the RIE electrode [60].

On PFOTS‐SU8 arrays, steady state was reached more slowly than on the N_2_‐SU8 Janus arrays. One possible explanation for the slow saturation for the PFOTS‐SU8 arrays is an adaptation of the silane coating to contact with water. The surface was not, however, permanently altered. After completing a series of drops, allowing the sample to dry, and neutralizing it for 5 minutes with an ionizer air blower, a second series of water drops resulted in the same drop charge‐versus‐number curve (Figure S1).

Drop charging on the APTES‐SU8 Janus arrays initially started positive, but after approximately 10 drops, the charges switched to negative. Thus, on our second hydrophilic surface, charges were separated at the rear contact line. The drop charge saturated at ≈‐0.1 nC (Figure 2b). This reversal of drop charge is consistent with the findings reported by Wong et al. [61] for flat surfaces which were partially coated with APTES. Wong et al. attributed the polarity flip to surface adaptation and the related change in surface chemistry. After prolonged exposure to air, the non‐polar hydrocarbon groups become more exposed. Exposing hydrocarbons is energetically favorable and leads to lower surface energy. When the surface is briefly exposed to water, the amino groups orient toward the surface due to their hydrophilicity. The first few drops are necessary to turn the amino groups and expose these polar groups. We would like to point out that Wong et al. used glass coated with a mixture of APTES and PFOTS or trichloro(propyl)silane. They added these hydrophobic compounds to increase the contact angles. On flat surfaces coated only with APTES water spreads and does not form drops, making charge measurements impossible prior to the Janus array fabrication carried out in this study.

### 2.3 Drop Charging Mechanisms

**FIGURE 3:**
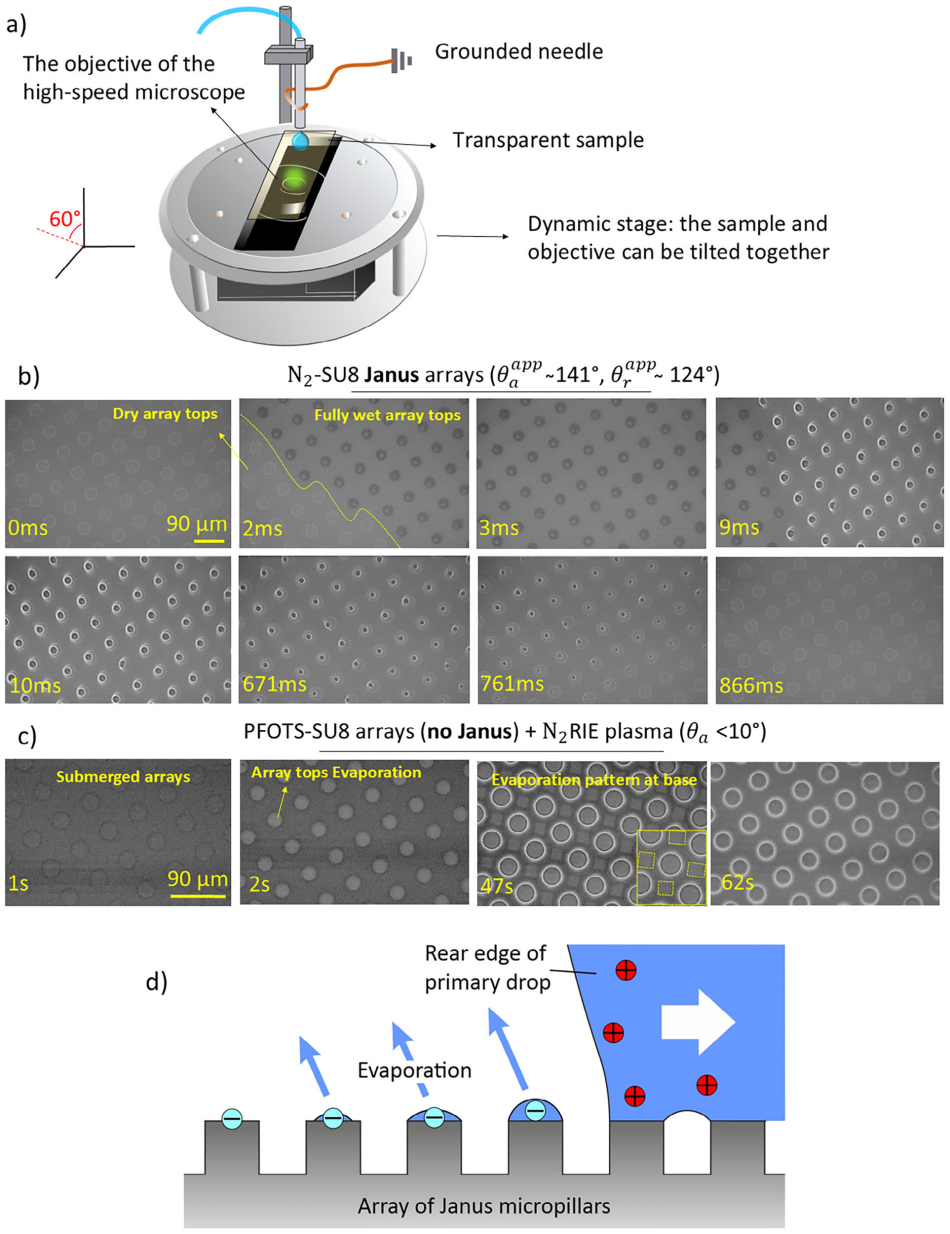
a) Schematic of the high‐speed reflection microscope used to image sliding water drops. b) Sequential images from high‐speed reflection microscopy video of a representative sliding drop. The video was recorded under the following sliding conditions: 45 ± 1 µL drop sliding down a 60° tilted surface on an N_2_‐SU8 Janus array at RH ≈ 35%. c) PFOTS‐SU8 arrays after N_2_ RIE plasma treatment (hydrophilized control). The drop spreads and immediately enters the Wenzel state (pillars submerged). Upon evaporation, the pillar tops appear brighter after ≈1 s; the liquid film between pillars evaporates over ≈40–50 s, and the surface becomes dry within ≈1 min. d) Schematic of the charge‐deposition mechanism for a receding drop sliding over an array of micropillars with hydrophobic walls and substrate and hydrophilic top faces.

To find out more about the charging mechanism, we recorded videos of drops sliding down superhydrophobic (PFOTS‐SU8 arrays) and Janus arrays using reflection microscopy. Our home‐built reflection microscope was equipped with a high‐speed camera. 45±1 µL water drops slid down the surface, which was tilted by 60°; a schematic of the main part of the setup is illustrated (Figure 3a). The full image of the actual setup is shown in Figure S3. When drops reached the field of view after ≈1 cm of sliding, they had a speed of ≈26 cm/s.

Figure 3b shows a sequence of images recorded when a drop passed over a N_2_‐SU8 Janus arrays from top right to bottom left. The surface had been neutralized prior to sliding, so this drop corresponds to the first drop in the serial measurements discussed above. At 0 ms, the drop had not yet reached the area imaged with the microscope; the image shows the array in air. At 2 ms the front of the drop had reached the field of view and water covered the top right part of the image. As a result, the top faces of the pillars appear darker because less light was reflected from the SU8‐water interface than from the SU8‐air interface. Within 3 ms, the drop covered the tops of all the arrays in the field of view. The reflection microscope images confirm that the drop is in the Cassie state. After 9 ms, the rear of the drop passes the field of view; only the left part is still underneath the drop. The bright rings observed around the rim of many pillar tops after the rear edge has passed are caused by satellite droplets deposited on the hydrophilic top faces (30 µm diameter). In our reflection microscope, dry SU8‐air regions appear bright, whereas wetted SU8‐water regions appear dark; a curved meniscus on a pillar top therefore produces a bright rim surrounding a darker wetted center. These satellite droplets evaporated within ≈0.8 s. Since the time interval between sliding drops was 4 s, every drop encountered a dry surface. Therefore, the proposed charge separation occurs at the moment of pinch‐off from the primary drop (millisecond timescale). Subsequent evaporation of the isolated satellite droplets cannot affect the charge balance of the primary drop. The satellite droplets may recede across the top of each array and undergo slide electrification, but this electrification does not contribute to the net surface charge on the top of the arrays because they ultimately evaporate.

Quantitatively, the deposited satellite droplets represent only a minute fraction of the primary drop. From the observed geometry of the satellite droplets on individual pillar tops, we estimate a typical volume of ≈2.6 pL (Supporting Information, Satellite droplet volume on the Janus sample). Summed over the 5 cm sliding distance used in the charging experiments, this corresponds to a total residual volume of ≈0.13 µL released from a single 45 ± 1 µL primary drop, i.e., only ≈0.3% of the initial drop volume. Thus, while the satellite droplets are numerous and mechanistically important for charge partitioning, the associated mass loss is negligible on the scale of the sliding drop.

On PFOTS‐SU8 arrays, satellite droplets‐like those seen on the Janus arrays, were not observed at all. However, very tiny droplet residues were observed at a single small point on each pillar (Figure S4). A deposition of liquid residues on superhydrophobic arrays of micropillars has been observed before [62, 63, 64].

For comparison, we exposed PFOTS‐SU8 arrays to N_2_ RIE plasma treatment so that they became hydrophilic (Figure 3c). Such samples were fully wetted. Drops immediately went to the Wenzel state, in which the pillars were submerged in water. After ≈1 s, the water had receded so that the tops of the pillars fell dry, causing them to appear brighter. After ≈40 seconds, the thin film of water between the arrays evaporated, leaving only menisci of water around every micropillar. Finally, the sample dried within a minute. Figure 3b,c visualize the contrast in drop behavior between the Cassie and Wenzel states.

In contrast to the flat PFOTS‐SU8 surfaces, charge separation on the Janus arrays was different and no satellite droplets were detected. As no charge transfer is possible between the primary and satellite droplet after separation, we conclude that the satellite droplets left on the arrays of micropillars are already charged. When the satellite droplets are shearing off at the rear of the primary drop, a slight excess of ions from the diffuse electric double layer may remain in the primary drop. We proposed that this imbalance leads to a net charging of the satellite droplets and, consequently, the formation of the countercharges in the primary drop. The charge deposition mechanism during drop sliding on a hydrophilic solid surface is illustrated schematically in Figure 3d. At the rear edge of a drop on a superhydrophobic surface, capillary bridges form between the drop and the pillar tops. As the drop moves, these bridges are stretched and eventually rupture [54, 65]. As a result, satellite droplets are left on the top faces.

The key difference to a planar, homogeneous hydrophilic surface is the absence of a laterally connected, conductive water film that could rapidly redistribute and neutralize charge over macroscopic distances. Isolating the hydrophilic tops by hydrophobic regions is expected to increase the resistance substantially compared with a fully wetted planar hydrophilic surface.

This satellite droplet pinch‐off/deposition pathway provides a plausible additional contribution to the net charge measured in Figure 2b on the Janus arrays. The RIE plasma interaction with PFOTS‐SU8 resulted in the formation of positive surface species, which is one reason for enhanced slide electrification, consistent with earlier work where increases on the order of ≈64% were reported [59]. In our case, the steady‐state charge on N_2_‐SU8 Janus arrays was three times larger compared with PFOTS‐SU8 pillar arrays. Beyond plasma effects, the enhanced charging is consistent with stronger charge separation on the hydrophilic top faces and may be aided by the satellite droplet‐mediated charge‐partitioning mechanism identified here.

The phenomenon of charge separation following the detachment of tiny satellite droplets from larger primary drops was reported more than 100 years ago. At that time, experiments were conducted to improve the understanding of cloud charging and the presence of electric fields near waterfalls. In 1914, Nolan [66] allowed drops to fall into a very strong horizontal air current. Each drop, as it entered the airstream, was immediately shattered. A mixture of small droplets of varying sizes was produced, including a small number of medium‐sized drops and a large number of exceedingly fine ones. The fine drops were negatively charged, while the medium‐sized drops gained a positive charge. A similar asymmetric charge distribution had also been observed when a water beam hits a target [67] and is atomized or when drops impact on a surface and splash [68, 69]. However, since the concept of diffuse electric double layers had not yet been established at the time, the results could not be properly contextualized. In addition, the secondary droplets were split off in a highly undefined way. Using arrays of Janus micropillars makes it possible to separate monodisperse secondary droplets from a larger primary drop and study the process more systematically.

### 2.4 Surface Characterization

Janus micropillar arrays are introduced here to enable controlled slide‐electrification measurements on hydrophilic contacts by maintaining Cassie‐state motion (high apparent contact angles) and suppressing discharge pathways associated with a laterally connected adsorbed‐water layer. The satellite droplet‐mediated pathway identified in Figure 3 further requires that the hydrophilic contact be confined to the pillar tops. We therefore perform surface‐sensitive characterization for two reasons: (i) to verify that the fabricated arrays are genuinely Janus, with PFOTS retained between pillars/sidewalls while the pillar tops are defluorinated/hydrophilized; and (ii) to identify the chemical terminations introduced by APTES and the plasma‐induced surface functionalities created by N_2_‐RIE on the top faces, since the polarity, magnitude, and saturation behavior in Figure 2 depend on this top‐face chemistry.

#### 2.4.1 Janus Micropillar Arrays

**FIGURE 4:**
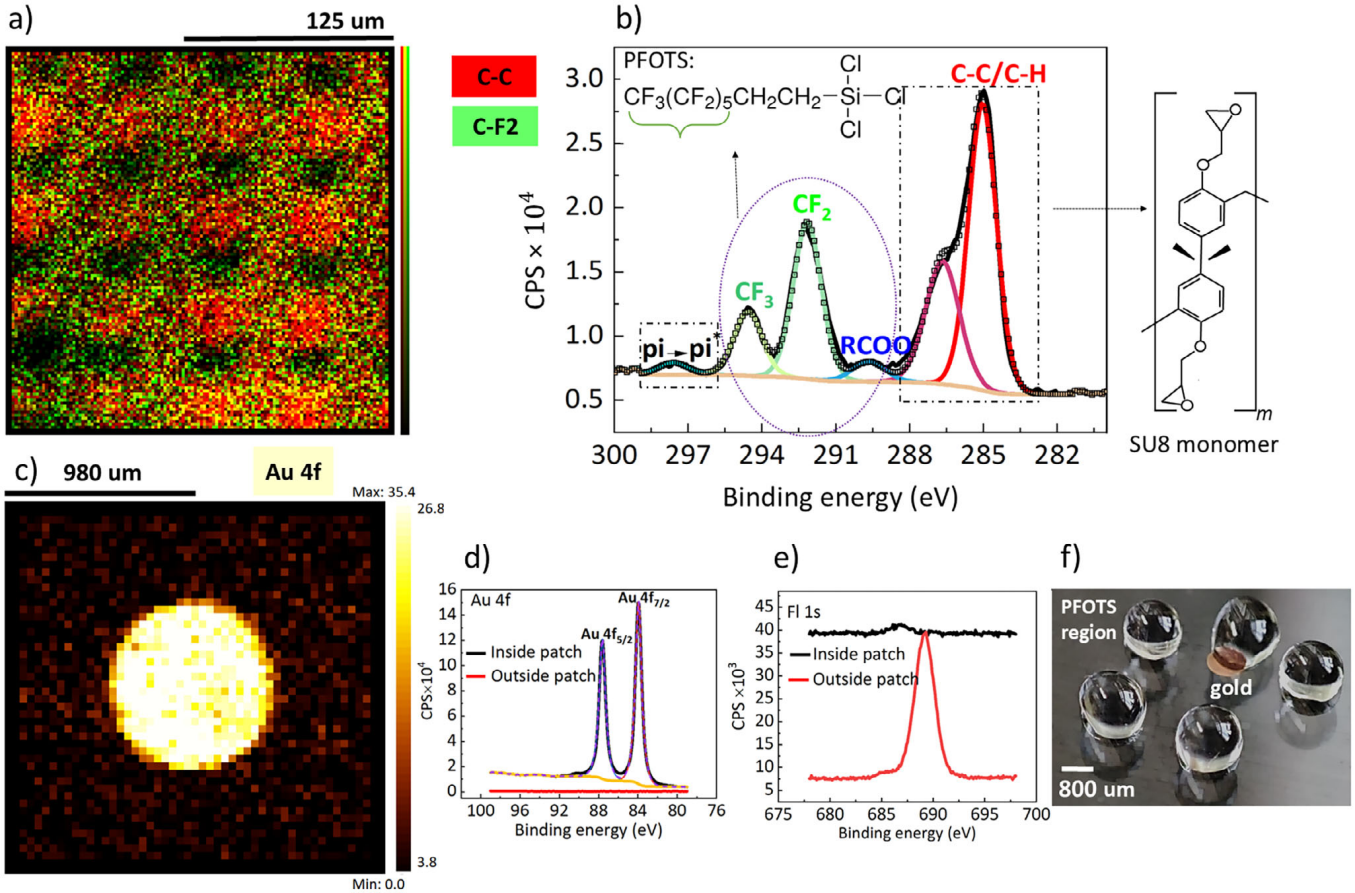
a) XPS imaging (256 × 256 µm^2^) of the N_2_‐SU8 Janus arrays; an overlay of two images representing the spatial distribution of photoelectrons at specific kinetic energies of 1201.6 and 1194.04 eV correspond to C─C and C─F_2_ bonds. The red regions represent C─C/C─H bonds, indicating the top of the arrays, while the green regions correspond to C─F_2_ bonds, indicating the substrate. b) High‐resolution spectra of the C 1s region of the Janus pillar arrays showing chemical groups of PFOTS and SU8: C‐C/C‐H, RCOO, CF_2_, CF_3_, and π‐π* transitions, reflecting the surface chemistry at both the top of the pillars and substrate. c) XPS snap map of Au 4f on the gold‐PFOTS patch surface. d, e) High‐resolution spectra of Au 4f and F 1s from inside and outside the gold patch, pass energy: 40 eV. f) Optical image of several drops deposited on fully hydrophobic (PFOTS) and gold‐PFOTS regions.

To verify that our fabrication process for the N_2_‐SU8 Janus arrays leads to fluorine‐free regions on top of the micropillars (which contain O‐ and N‐functional groups) and fluorine‐containing regions on the PFOTS‐glass between pillars and PFOTS‐SU8 sidewalls, we carried out XPS imaging. XPS provided spectral and spatial data to differentiate elements and chemical states of N_2_‐SU8 Janus arrays. First evidence is obtained by spatially resolved images recorded at 1201.6 eV and 1194.04 eV kinetic energies (Figure 4a). The two energies corresponding to C─C/C─H bonds (binding energy: 285.0 eV, red, pillar tops) and C─F_2_ bonds (binding energy: 292.3 eV, green, substrate) were selected for imaging. The overlay of the two images shows that C‐F_2_ is primarily present between the pillars and not on top of them (where PFOTS was removed by the plasma), confirming that PFOTS was successfully retained on the substrate.

XPS spectra were collected from areas of 700 × 300 µm^2^; high‐resolution spectra of O 1s and C 1s core‐levels are shown in Figure 4b and Figure S5, respectively. The C 1s region detected over that area of surface revealed PFOTS peaks alongside SU8 peaks. Three peaks at 285 eV, 286.5 eV, and 297.6 eV were characteristic of SU8 [70]. The typical PFOTS spectrum contains two hydrocarbon peaks (285, 286.5 eV), which here overlapped with SU‐8, and two fluorocarbon peaks (CF_2_ at 292 eV and CF_3_ at 293 eV) and a small peak at 290 eV, which is commonly observed in PFOTS [59, 71, 72], FOTS [73]. Based on the ability of XPS spectral and spatial data to chemically distinguish SU8 from PFOTS, we conclude that the Janus micropillar arrays were fabricated such that CF_2_, the main fluorocarbon component of PFOTS, was selectively removed from the tops of the pillars while being retained at the substrate.

Dark areas are observed in Figure 4a. To demonstrate that these dark areas do not indicate C‐C/C‐H and CF_2_ depletion, but rather result from a shadowing effect, we conducted control experiments in which the micropillar height was completely removed, yielding a flat patch configuration (gold‐PFOTS).

***Gold‐PFOTS Patch***. We performed XPS mapping on a gold‐PFOTS patch fabricated using the same method as the Janus arrays. In the gold patch (diameter: 800 µm), which was level with the fluorinated zone, no shadowing was observed when both regions were at the same height (Figure 4c). The chemical mapping image from the Au 4f core‐level (FOV: 2 × 2 mm^2^) was constructed by stacking multiple images acquired in the binding energy range of 96.6–76.5 eV with a step size of 0.2 eV. According to the color scale bar, the white and bright yellow pixels, representing an intense signal, indicated that Au was present only within the patch.

To examine PFOTS retention outside the gold patch, the Au 4f and F 1s core‐level spectra were compared inside and outside the patch. In the Au 4f spectra, inside the patch showed characteristic Au doublet peaks at Au 4f_7_/_2_: 84.0 eV and Au 4f_5_/_2_: 87.7 eV (Δ = 3.7 eV), while no Au signal was observed outside (Figure 4d). In the F 1s spectra, a large signal at 687–690 eV attributed to organic fluorine [74] originating from PFOTS outside the patch, with only a negligible fluorine signal inside, originating from SU8 photoinitiators [75] (Figure 4e). An optical image of the sample with a few drops outside the gold (adv: 115°, rec: 95°) confirmed that the hydrophobicity provided by PFOTS was successfully preserved and repelled the drops (Figure 4f). A single drop was placed across both areas, and its contact line was influenced by the gold surface, which has a higher surface energy than PFOTS.

We conclude that the shadowing occurred due to the pillar height. The fabrication method of Janus arrays safely and effectively retains PFOTS stability. Mammen et al. [32] showed that retaining PFOTS in the Janus arrays is key to maintaining the drop in the Cassie state.

#### 2.4.2 Surface Chemistry of Hydrophilic Janus Array Tops Surfaces

**FIGURE 5:**
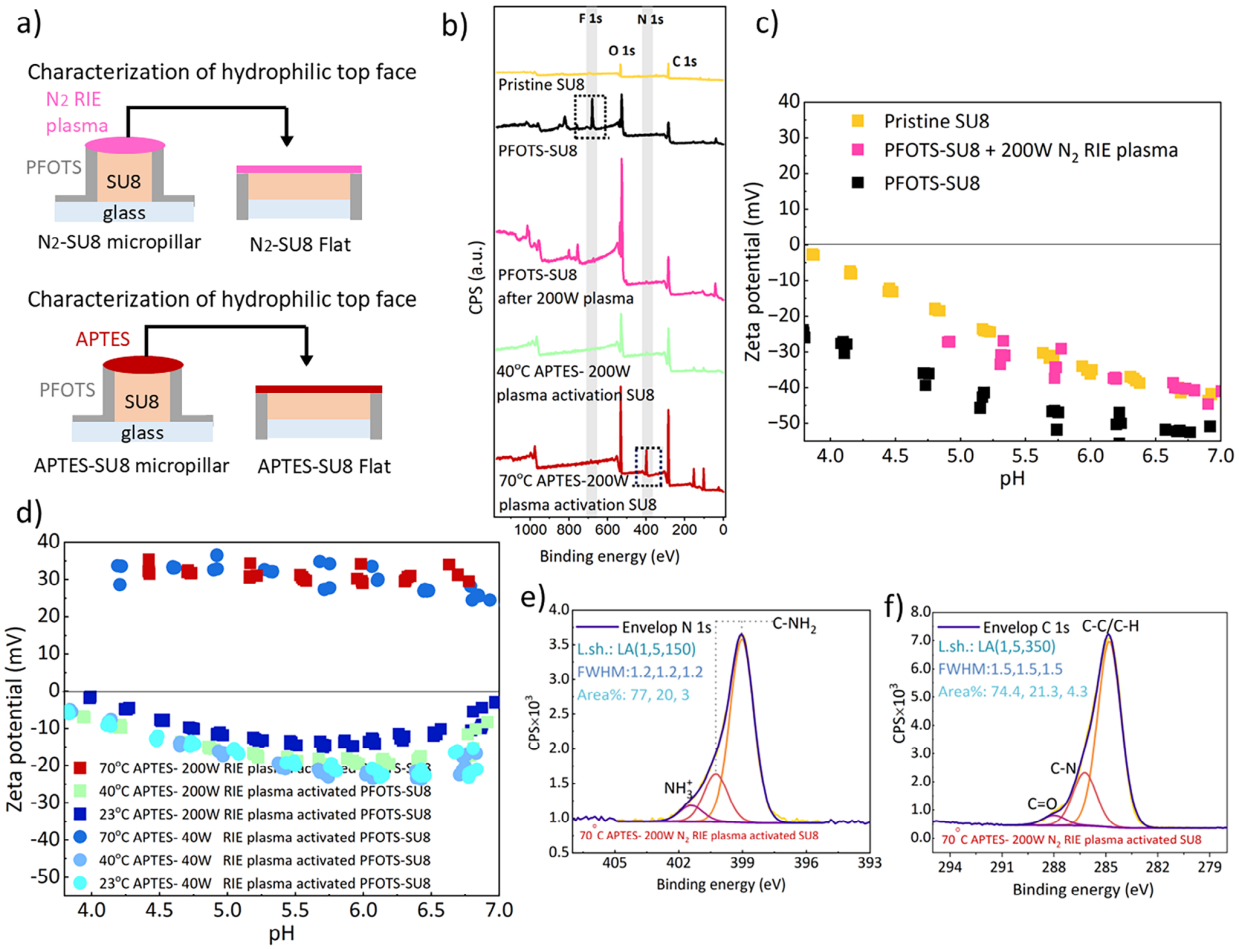
Characterization of hydrophilic Janus array tops via their flat surfaces. a) Hydrophilization using N_2_ RIE plasma and APTES vapor deposition. b) Survey spectra recorded at each preparation step. Zeta potential of c) pristine SU8, and PFOTS‐SU8 before and after N_2_ RIE plasma, d) thermally activated APTES deposited on N_2_ RIE plasma‐activated PFOTS‐SU8 surfaces at various CVD temperatures and plasma powers. e,f) Curve fitting of the C 1s and N 1s regions for APTES deposited at a CVD temperature of 70°C and a plasma power of 200 W.

To understand how the hydrophilic tops of the Janus micropillars increase the drop charge on N_2_‐SU8 arrays and lead to negative charge saturation in APTES‐SU8 arrays, a knowledge of surface chemistry is required. For this reason, we applied XPS and zeta potential measurements to flat SU8 surfaces, which were chemically treated like the top faces of the N_2_‐ and APTES‐SU8 Janus arrays (Figure 5a).

***N_2_‐SU8 Flat Surface***: The XPS survey of pristine SU8 showed dominant peaks at the C 1s and O 1s core‐levels, and a weak signal at F 1s core‐level from the SU8 photoinitiator (Figure 5b, yellow). After fluorosilanization, a strong peak in F 1s core‐level around 681 eV confirmed PFOTS chemisorption (Figure 5b, black), shifting to a lower binding energy, likely caused by charging. N_2_ RIE plasma etched PFOTS from SU8, so the peak in the F 1s region disappeared, while the peak intensity in the O 1s region increased (Figure 5b, pink) and NH_3_^+^/NH_4_^+^ species appeared in the N 1s region at 401.0/402.5 eV [76, 77, 78] (Figure S6a), indicating successful functionalization (Figure 5b, pink). As noted, in RIE reactors, samples are mounted on the power electrode of the reactor and bombarded with positive ions.

Zeta potentials of PFOTS‐SU8 surfaces were the most negative over the whole pH range (‐60 to ‐50 mV at pH 6.5–5.5) (Figure 5c, black). After N_2_ RIE plasma, it increased by≈ 12 mV (‐40 to ‐30 mV) (Figure 5c, pink), likely due to fluorine removal and the formation of positively charged species.

Sliding drops on PFOTS‐SU8 arrays, which had a hydrophobic top surface, acquired a positive charge. After hydrophilization of the tops using N_2_ RIE plasma, the drop charge increased threefold. Similarly, a 64% increase in the drop charge, accompanied by a less negative zeta potential, was observed on fluorinated glass after plasma treatment near the power electrode (different reactor) as reported in our previous study [59].

***APTES‐SU8 Flat Surface***: A separate Janus micropillar was rendered hydrophilic via APTES functionalization. Here, we focus on how this functionalization alters the surface chemistry, aiming to elucidate the origin of negative drop electrification.

For an effective APTES deposition, it was important to apply a temperature of 70°C [79, 80]. Thermally activated APTES‐vapor deposition on SU8 was studied at different substrate temperatures (23°C ±1, 40°C ±1, 70°C ±1) in the CVD, and different N_2_ RIE plasma powers (200 W, 40 W) for SU8 activation.

Deposition at 40°C increased the nitrogen peak slightly as compared to N_2_‐SU8, PFOTS‐SU8 and pristine SU8 samples (Figure 5b green vs. pink, black, yellow), indicating poor APTES coverage. Correspondingly, the zeta potentials were negative, between 0 and ‐20 mV (Figure 5d). However, deposition at 70°C and 200 W showed a significant peak at the N 1s core level, whereas at 40 W only a weak peak was observed (Figure 5b, red vs. green). The surface prepared at this high temperature showed a zeta potential in the range of +30 to +40 mV, which is at least 40 mV higher than that of samples deposited at 40°C and 23°C (Figure 5d). At a constant 23°C, APTES deposited on activated SU8 with higher plasma power (200 W vs. 40 W) showed a higher zeta potential, for example, 11 mV higher at pH 6.5, despite both samples having identical CVD conditions. The evolution of APTES coverage as a function of CVD temperature is shown in Figure S6b, confirming increased APTES coverage at 70°C.

The strong positive zeta potential originated from the dissociation of amine groups; to investigate this, the high‐resolution scan of the N 1s and C 1s regions was analyzed (Figure 5e,f). It revealed an overall increase in all nitrogen environments with the introduction of the APTES precursor. At room temperature and low plasma power, a small increase in protonated nitrogen species was evident in the XPS spectra [76]. Increasing the temperature to 70°C, and operating at a higher power (200 W vs. 40 W), a significant increase in primary amine concentration was detected. This was observed by both a large signal at 399.2 eV in the XPS, and the presence of the N‐H bending mode (Figure 5e; Figure S6c) of primary amines at 1600 cm^−^^1^ detected by GA‐FTIR (Grazing Angle FTIR). Further support for the formation of this moiety was found in the form of emission intensity at 286.2 eV in the XPS (Figure 5f), consistent with C‐N environments.

Although NH_3_^+^ had previously been observed in the N_2_‐SU8 XPS scans, this peak intensity further increased following APTES deposition, indicating these molecules may be attributed either to plasma‐SU8 interaction, or to proton transfer from the relatively acidic ‐OH groups on the surface to the amino groups in the silane moieties [76]. This process may be facilitated by the presence of adsorbed water, which enhances the transfer of protons from silanol groups to the amino groups within the silane molecules. The CVD chamber reached the desired condition 5–6 minutes after the start of the reaction. It was evacuated to a base pressure of 60 mbar in ≈4 minutes, facilitating the presence of water molecules on the N_2_‐SU8. Following evacuation, the CVD chamber was heated, reaching 70 °C in 2–3 minutes.

In drop charging (Figure 2b), the negative drop charge observed at steady state (‐0.1 nC), which was previously correlated with a positive zeta potential (+30 to +40 mV, Figure 5d), is further supported by the presence of primary amine groups (‐NH_2_). These groups became protonated (‐NH_3_^+^) upon contact with water (with low conductivity). Thus, the magnitude and sign of the drop charge can be dictated by the hydrophilization method, which determines the surface chemistry. This is demonstrated by the charge‐enhancing effect of N_2_ RIE plasma and the negative polarity induced by APTES.

The peaks at 102.5 ± 0.5 [81, 82] eV in the Si 2p (Figure S6d) arise from organic silicon atoms in the APTES moieties, which are bonded to the surface and/or to other APTES molecules via Si‐O‐Si linkages (siloxane), not from inorganic silicon in the underlying glass (which may not be observed due to electronic inelastic transport pathways). Since the SU8 layer (≈12 µm thick) is much thicker than the X‐ray penetration depth, the observed Si photoemission is attributed to APTES. An increase in the peak area of siloxane was observed with higher temperatures (23°C, 40°C, 70°C). The peaks at: 1000–1130 cm^−1^ in the GA‐FTIR spectrum (Figure S6c) confirm siloxane [83] bonds between N_2_‐SU8 molecules and/or APTES, confirming the presence of covalent bonding [84]. The samples were rinsed and evacuated after reaction to remove unbound chains. Studies have shown that increasing the temperature from room temperature to 70°C increases the APTES thickness up to 1 nm [79, 80].

The small peak at 288.0 eV corresponding to C═O groups at the C 1s (Figure 5f) suggests that, in addition to OH, C═O may also contribute to surface chemistry. Consequently, due to hydrogen bonding with some of APTES, an additional species appears at around 400.0 eV, which we ascribed to carbonyl‐protonated amine groups, i.e., hydrogen‐bonded pairs.

We characterized fabricated Janus arrays with hydrophilic tops and a hydrophobic substrate and walls. XPS imaging confirmed the chemical contrast; N_2_ RIE plasma removed the fluorinated groups from the tops while they were retained on the substrate. On the hydrophilic tops created by the plasma, positively charged molecules formed in addition to defluorination. These are attributed to reduced negative zeta potential and increased drop charge compared with PFOTS‐SU8 arrays. The increased charge was even more than in the flat PFOTS‐SU8 sample in the steady state. In contrast, alternative hydrophilization created by APTES deposition introduced NH_2_ groups, which may explain the observed reversal in drop charging. We demonstrate how the top‐surface chemistry controls drop charging.

## 3 Conclusions

We introduce Janus micropillar arrays in which the tops of the micropillars are hydrophilic (advancing contact angle θ_adv_ < 10°), while the sidewalls and substrate are rendered hydrophobic. The arrays cover an area on the order of several square centimeters (≈ 7 cm × 2.5 cm) which are large enough to study sliding drops. Water drops placed on the arrays adopt the Cassie state with apparent advancing and receding contact angles of around 140° and 122° respectively.

Using these Janus micropillar arrays, we demonstrate that hydrophilic interfaces generate spontaneous charge separation in sliding water drops. However, this mechanism differs fundamentally from slide electrification onflat surfaces. High‐speed reflection microscopy revealed that satellite picodroplets are deposited on every pillar top at the receding edge. Charge separation occurs within the liquid phase during picodroplet pinch‐off and deposition, rather than arising continuously at the receding contact line. The picodroplets evaporate within 0.2 – 0.8 s. As a consequence, the deposited charges and the primary drop carry opposite net charges after separation.

XPS chemical imaging and contact angle measurements confirm successful Janus fabrication. The polarity and magnitude of the drop charge are governed by surface chemistry. Hydrophilization of the Janus pillar tops by N_2_ RIE plasma enhances the drop charge beyond that on flat PFOTS, as the plasma‐PFOTS‐SU8 interaction forms positively charged species and etches PFOTS. Hydrophilization by APTES at 70°C inverts the polarity and yields a negative steady state drop charge, consistent with the high density of primary amines and the strongly positive zeta potential.

## 4 Experimental Section

### 4.1 Fabrication of Janus Micropillar Arrays

**FIGURE 6:**
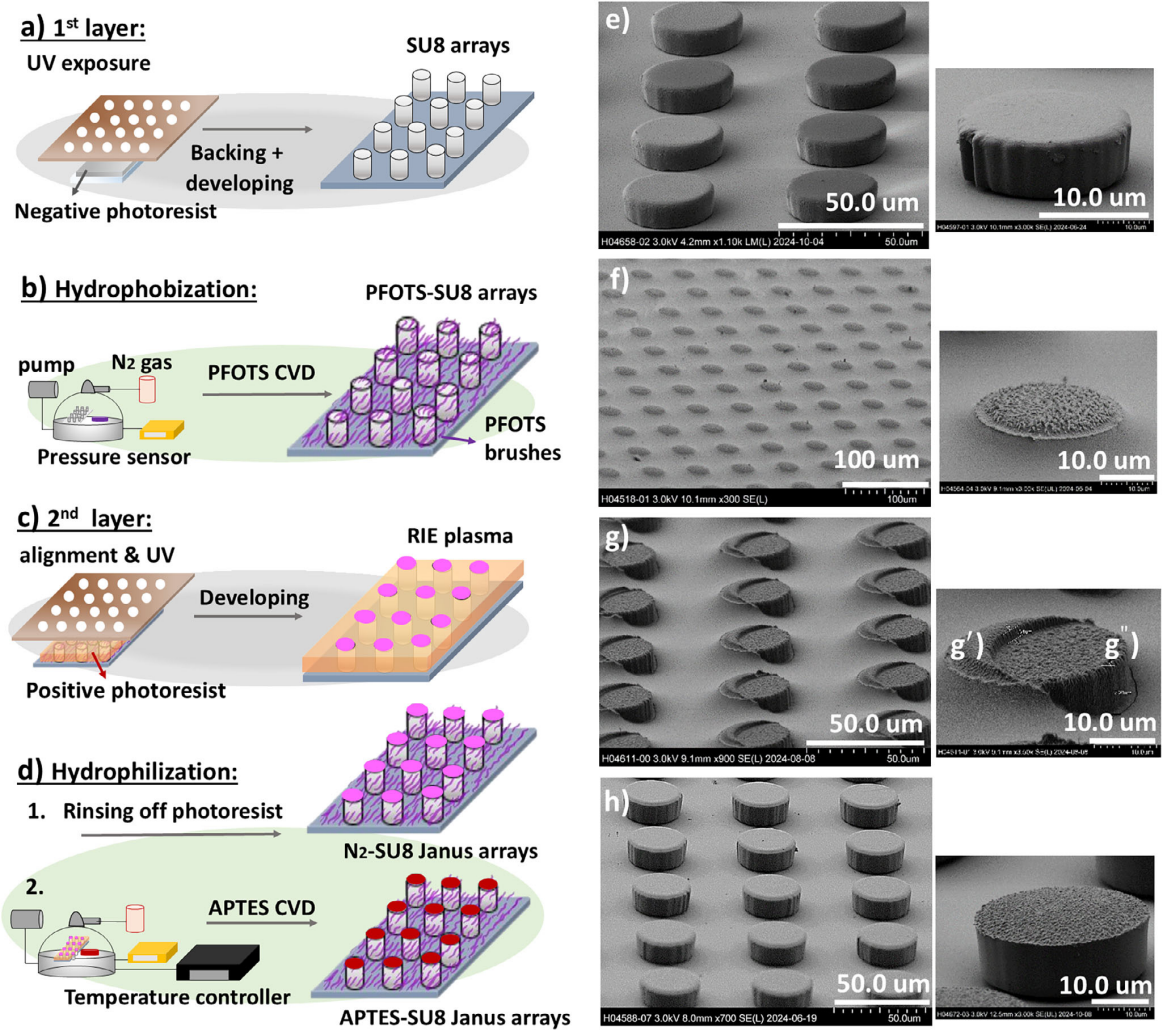
Schematic showing step‐by‐step fabrication of Janus micropillar arrays with hydrophilic tops and hydrophobic side‐walls and substrate (a–d). SEM side‐view and close‐up images of single micropillars highlight the hydrophilic top surface (e–h). a) First layer: SU8‐arrays fabricated via photolithography (e, corresponding SEM image). b) PFOTS vapor deposition to make the SU8 hydrophobic. c) Second layer: photolithography of the positive resist over the SU8‐arrays. Following photomask alignment and development, N_2_ RIE plasma was applied (f, SEM image). The dark circular patterns on top of the pillars resulted from wet and dry etching, while the substrate and side‐walls of the pillars were masked by the second layer. Shifting the photomask in X‐ or Y‐axis after alignment (g, SEM image). The g^’^ is the undissolved area after development, and g^”^ is the dissolved area. The roughness near g^”^ is the effect of the post‐plasma etching. d) APTES‐SU8 Janus arrays (h, SEM image): The tops of micropillars (dark part of image, f) which were already activated using N_2_ RIE plasma, were also modified by APTES. Then, the second layer (bright area of the image, f) was removed.

***First Layer*** (Figure 6a,e): In a cleanroom 4‐inch soda‐lime glass substrates (1 mm thick) were ultrasonicated (10 min) in acetone and 2‐propanol followed by N_2_ drying and O_2_ plasma cleaning (180 W, 10 min, 0.3 mbar, Diener, Germany). An ionizing N_2_ gun (Simco‐Ion's AirForce) was used for drying and to remove microscopic fibers and dust. The substrates were then de‐moisturized by heating to 70°C for a few minutes. 1–3 mL of SU8 GM1060 negative‐tone photoresist was dispensed onto the clean glass substrates using a pipette and spin‐coated at 500 rpm for 5 seconds, then ramped to 2000 rpm for 40 seconds and 3000 rpm for 2 s. After spin coating, the samples were soft‐baked on a hotplate at 65°C for 30 minutes, followed by 95°C for 10 minutes, and then 65°C for another 30 minutes to reduce residual solvent. After cooling, samples were exposed to UV light on a Mask aligner (MA 6 SUSS MicroTec) through an anti‐reflective chrome on soda lime photomask (exposure time 5 s, UV intensity 10 mW/cm^2^, I‐line at 365 nm, soft contact 25 µm gap). To produce SU8 micropillar arrays (briefly, *SU8*‐*arrays)* the photomask was designed with circular arrays, each circle having a diameter of 30 µm and spaced 30 µm apart. The surface fraction was φ = 0.2. For flat surfaces (*SU8 flats*), the same process was used, but with plain glass (no chromium) instead of a photomask to create. All samples after UV exposure were then baked at 65°C for 30 minutes, ramped to 95°C, and baked for another 2 minutes. To dissolve uncross‐linked photoresist (the unillumined part) the samples were developed (micro resist, Germany) and rinsed with 2‐propanol. Finally, the desired arrays of micropillars formed and were hard‐baked at 150°C for 2 hours with slow cooling down to room temperature.

***Hydrophobization*** (Figure 6b): Afterward, the flat samples and SU8‐arrays were O_2_ plasma cleaned (180 W, 5 min, 0.3 mbar, 40 KHz) and immediately transferred to a desiccator for silanization with PFOTS (97%, #CAS 78560‐45‐9, Merck, Germany). This silane was selected for its low boiling point (180°C), facilitating easy evaporation, and its high reactivity. Silanization was performed in a chemical vapor deposition (CVD) chamber (25 cm diameter, V = 9.2 L). Under a nitrogen stream, **0.5 ± 0.1 mL** of PFOTS was added to a glass Petri dish inside the chamber, and samples were placed at the ca. 5 cm from the Petri dish. The chamber was evacuated to 59 mbar. To reduce moisture on the chamber walls and ensure consistent pumping time, the desiccator was evacuated and vented with N_2_ gas before starting the reaction. The PFOTS deposition was most efficient when the ambient humidity was low (⩽ 30%) [85]. To lower CVD's dependence on environmental humidity, N_2_ gas (99.999%) was then purged [85] into the chamber, raising the chamber pressure to 140 mbar. The samples were under reaction for 35 minutes. Finally, the fluorinated samples were rinsed with ethanol and evacuated at mid‐vacuum (0.1 mbar) to remove unbonded silanes. We call these samples *PFOTS‐SU8 flats* and *PFOTS‐SU8 arrays*, respectively.

***Second Layer*** (Figure 6c,f,g): To make only the top faces of PFOTS‐SU8 arrays hydrophilic, 1–3 mL of positive‐tone photoresist (AZ 10XT, MicroChemicals, Germany) was dispensed using a pipette on the arrays. Spin coating was performed in two steps: first at 500 rpm for 5 s (200 rpm/s), then ramped to 3000 rpm for 40 s with the same acceleration. The samples were annealed at 95°C for 2 minutes. Water was an indispensable precursor for the photochemistry of the resists. To allow the resist to rehydrate, samples were left in air for 15–20 minutes [86]. The same mask used to produce the SU8 arrays was loaded onto the mask aligner and aligned over the SU8 micropillars to ensure precise pattern matching (an example of an aligned sample as a few mm‐scale image was provided in Figure S7).

To align the photomask and sample patterns, eight alignment markers (5 µm crosses) were placed at the edges and corners of the photomask. Complete alignment means that each micro‐circle on the photomask precisely overlaps with the underlying array of micropillars. In the complete alignment, details of layers and sensitivity of the process were not visible since the two layers overlap. Any shift, less than the micropillar diameter, as deliberately introduced upon alignment of the sample in Figure 6g, showing how micropillars become obscured beneath the second layer. This was not visible in the perfectly aligned case (Figure 6f).

After alignment, the samples were exposed to UV light for 8 s (with conditions similar to those used in the SU8‐arrays step). UV‐exposed areas were soluble in the photoresist developer (Figure 6c,f). After development, the sample was rinsed with Milli‐Q water and dried with the N_2_ gun. Next, the top‐opened micropillars became hydrophilic through N_2_ RIE plasma (200 W, 120 s, Nordson MARCH, USA). This power of plasma was chosen to remove PFOTS and residues from the 2^nd^ layer. Heating the sample during RIE plasma was kept low.

At this stage, the samples were classified into two groups: (Figure 6d‐1) The array of Janus micropillars with hydrophilized tops via N_2_ RIE plasma, referred to as the *N*_*2*_‐*SU8 Janus arrays*. In this group, the second layer was removed, and the samples were dried using an N_2_ gun before being sent for characterization. The PFOTS did not dissolve or decompose upon removal. In the second group (Figure 6d‐2), the sample with the second layer retained was thoroughly rinsed with Mili‐Q water after the plasma etching and subsequently placed in a desiccator for silanization using APTES (98%, #CAS 919‐30‐2, Merck, Germany).

***APTES Deposition***: After drying, the samples were placed in a CVD desiccator equipped with a temperature controller, which was connected to a plate inside the chamber using vacuum‐compatible components. Under a nitrogen stream, 2 mL of APTES was added to a glass Petri dish inside and then evacuated to 59 mbar with a membrane pump, which took approximately 3.5‐4 minutes. N_2_ gas (99.999%) was purged into the system until the internal pressure reached 140 mbar, as recorded by the pressure sensor. The stage plate holding the samples and the Petri dish was maintained at 23°C, 40°C, and 70°C during the reaction. Hereafter, all temperatures refer to the temperature of the plate that was inside the desiccator during CVD. After completing the reaction time (50 minutes), the samples were then rinsed, dried using an N_2_ gun, and placed in a 0.1 mbar mid‐vacuum chamber for 30 minutes to remove unbonded silane (Figure 6h). We call these samples APTES‐SU8 Janus arrays.

### 4.2 Methods

***Scanning Electron Microscopy***. Inset SEM images were captured using a Hitachi SU8000 instrument with an accelerating voltage of 3 kV. All samples were loaded on a 70° tilted pin stub. Samples were sputtered with a Pt target using Ar plasma at a current of 35 mA, yielding a 10 nm Pt layer. To ensure uniform coating, each sample stub was rotated around its own axis in addition to the rotation of the stage during sputtering (Safematic, Germany).

***Plasma RIE***: We used a CCP plasma etcher with 200 W, 40 W forward power, 0 reflected power operated at a frequency of 13.56 MHz. The base pressure and process pressure, were 55 Pa and 90 Pa. 90 sccm of ultra‐high purity N_2_ (99.99999%) was injected as the working gas.

***Water Contact Angles***: Static advancing and receding contact angles of water were measured with a goniometer (Krüss DSA100E, Germany). Side view videos of sessile drops were recorded during inflation and deflation (2.7 µL s^−1^) between 6 and 60 µL using a Hamilton syringe (inner diameter: 0.24 cm). Contact angles were determined by fitting an ellipse model for pillars and flat surfaces to the contour images. Each data point was the average of at least three individual measurements on different areas of the surface.

***Drop Charge Measurements***: To measure the charge of sliding water drops, samples were mounted on a grounded metallic plate tilted at 60°. Residual surface charges were neutralized for 10 minutes using a corona discharge ionizer (Simco‐Ion, Aerostat PC, USA). MilliQ water drops (45±1 µL, 18.2 MΩ cm resistivity) were dispensed from a grounded stainless steel needle Hamilton syringe via a peristaltic pump (Gilson Minipuls 3). The drops, separated by 4 seconds, detached from the needle (5‐8 mm above the surface) and slid down the plate after landing. Upon sliding, the drops first contacted a grounded electrode and then traveled 5 cm further to a second electrode connected to a low‐noise current amplifier (response time: 0.8 ms, DDPCA‐300, FEMTO). Upon reaching drop to the second electrode, the drop discharges through the amplifier input, producing a transient current *I*(*t*). The drop charge reported in Figure 2b was calculated as Q=∫I(t)dt over the full transient (0.01–1.5 ms after first contact), after subtracting the baseline signal measured immediately before drop arrival. The first grounded electrode defines a common electrical reference and removes any residual charge carried by the drop prior to the final 5 cm sliding segment used for charge quantification.

***Reflection Microscope***: We performed bottom‐view imaging of the contact line dynamics and of the wetting state during drop sliding (Figure 3a). We used an inverted epifluorescence microscope (Olympus IX83) operated in reflection mode with a 20x objective (Olympus UCPLANFL N 20x) and a high‐power LED (Thorlabs DC2200, 525 nm). An objective inverter (InverterScope, LSM TECH, USA) was added to rotate the sample and objective simultaneously and reach a controlled tilting angle. Both the metallic stage and the needle used to create the drops were grounded. Bottom‐view images of the surface during drop sliding were acquired at 1000 fps using a high‐speed camera (Photron, Phantom TMX 7510).

***X‐ray Photoelectron Spectroscopy***: XPS was used for imaging and spectroscopy of the micropillars and flat surfaces. Regarding micropillars (Figure 4a), parallel imaging was performed using a Kratos Axis Supra, where the intensity of signal from a specific kinetic energy was collected over a 250 × 250 µm^2^ sample area across a two‐dimensional field of view and projected onto a two‐dimensional delay‐line detector (DLD) [87]. X‐ray powers of 300 W and 225 W, and pass energies of 160 eV and 40 eV were used for imaging and spectroscopy, respectively. On the gold‐PFOTS patch surface (Figure 4c), the gold mapping was carried out using a Thermo Nexa system with a spot size of 10 × 5 µm^2^, in a high‐resolution scan of Au 4f and F 1s at a pass energy of 40 eV. For flat samples (Figure 5), spectra were acquired (Kratos Axis Ultra DLD, UK) at a pass energy of 20 eV. All measurements employed a monochromatic Al Kα X‐ray source (hν = 1486.6 eV). The systems were calibrated to Au 4f = 83.95 eV. Shirley background subtraction was applied to all spectra. The high‐resolution scan of the Janus pillar spectra was calibrated to the C‐C/C‐H peak at 285.0 eV, while spectra from the flat samples were calibrated to the same peak at 284.8 eV. All data processed by CasXPS version 2.3.27PR1.7.

***Zeta Potential***: The zeta potential of flat surfaces was determined from streaming potentials and streaming currents using an electrokinetic potential analyzer (SurPASS 3 Anton Paar, Austria) over a macroscopic sample area of 20 × 10 mm^2^. The conductivity of the electrolyte solution was adjusted with 0.001 mol/L KCl dissolved in ultrapure water (resistivity: 18.2 MΩ·cm). For pH titration, 0.05 mol/L HCl and 0.05 mol/L KOH were used as acidic and basic solutions, respectively. Prior to each measurement, the pH probe was calibrated using three buffer solutions with pH values of 3, 7, and 10.

## Author Contributions

F.D.: Conceptualization, fabrication and experimentation, data processing, visualization, and writing. M.I.: Conducted and reviewed the XPS part and helped interpret results. S.S.: Assisted in numerical calculations, analyzing the results, visualization, scientific discussion, and writing. L.D.: Performed reflection microscope measurements, scientific discussion. H.‐J.B.: Supervised the project, planned the research, and helped interpret results and writing.

## Conflicts of Interest

The authors declare no conflicts of interest.

## Supporting information




**Supporting File**: smll73053‐sup‐0001‐SuppMat.docx.

## Data Availability

The data that support the findings of this study are available from the corresponding author upon reasonable request.
