# Supplementary material for: Water Drops Sliding Over Arrays of Janus Micropillars With Hydrophilic Tops: A New Mechanism of Drop Charging
Source: Small. 2026 Mar 11;22(36):e11728. doi: 10.1002/smll.202511728 (PMC13307243; doi:10.1002/smll.202511728)
Supplement: Supplementary file 1 — Supporting File: smll73053‐sup‐0001‐SuppMat.docx. [file SMLL-22-e11728-s001.docx]

**Water drops sliding over arrays of Janus micropillars with hydrophilic tops: A new mechanism of drop charging**

**Supporting Information**

Fahimeh Darvish*^1,2^, Mark Isaacs^3,4^, Sajjad Shumaly^1^, Lea Delance^1^, Hans-Jürgen Butt^1^

^1^Max Planck Institute for Polymer Research (MPI-P), Ackermannweg 10, 55128 Mainz, Germany

^2^Institute for Condensed Matter Physics, Technische Universität Darmstadt, Darmstadt, Germany

^3^HarwellXPS, Research Complex at Harwell, Rutherford Appleton Lab, Didcot OX11 0FA, United Kingdom

^4^Department of Chemistry, University College London, 20 Gower Street, London, WC1H 0AJ, United Kingdom

Table 1 lists Janus micro/nanopillars fabricated over the past two decades, along with their fabrication methods, research aims, and sample sizes. However, none of them reported drop sliding over their structures on the centimeter scale. The largest reported Janus structure we are aware of covered 100 mm^2^.

**Table1***:* Overview of fabricated Janus micro/nanopillars*.*

| **Schematic Directional contrast of Janus arrays** | **Method\** **Reported Janus arrays area from characterization data** | **Application\Aim** |
| --- | --- | --- |
| superhydrophobic all sides:  FAS-17 on the planted ZnO | 3D-printing + 2-steps chemical treatment  \up to 10 mm^2^ | Develop a Janus method for fog harvesting[1] |
| Hydrophobic side and substrate:  PFOTS on SU8  Hydrophilic side: Silica | Photolithography+ 3-steps chemical modification  \up to 200 μm^2^ | Demonstrate a Janus fabrication process[2] |
| Epoxy-based  polymer  Deposition of Au-Pd alloy on the epoxy-based polymer | Soft lithography, metallization  \a few tens of mm^2^ scale | Demonstrate a Janus fabrication process [3] |
| Pt, Au or Al on the polyurethane  acrylate (PUA)  Polymer | Photo and Soft- lithography+ 2-steps chemical modification | Demonstrate a Janus fabrication process [4, 5] |
| RGD polypeptide  on the deposited Au  Hydrophilic  side: Polyethylene glycol on the PDMS | Soft lithography + 3-steps chemical modification  \up to 100 μm^2^ | Develop a Janus method for the cell migration[6] |
| Hydrophilic side:  16-Mercapto exadecanoic acid  on Au  hydrophobic side: PFS on Si elliptical pillar | Soft lithography + 4-steps chemical treatment  \a few tens of mm^2^ scale | Demonstrate a Janus fabrication process[7] |
| Hydrophilic side and substrate: 16-Mercapto exadeca-noic acid on Au  Hydrophobic/philic side and substrate : poly (N-isopropylacrylamide) on Si elliptical pillar | Soft lithography, 4-steps chemical modification-\few ten mm^2^ scale up to 7 mm^2^ | Develop a Janus method for manipulating microfluidic motion[8] |
| hydrophilic top  ( ̴80°): Un-etched  hydrophilic base  ( ̴42°): RIE etched  Hydrophilic side: Polyethylene glycol on the PDMS | Electron beam lithography+ no chemical modification  \Up to 5.4 ×9.9 mm^2^ | Demonstrate a Janus fabrication process[9] |
| hydrophilic top:  N_2_ RIE plasma or  APTES  Hydropbobic side  and substrate:  PFOTS | Photolithography +  1 or 2-steps chemical modification  **\7 ×2.5 cm^2^** | Develop a Janus method for **sliding drop electrification** (this work) |


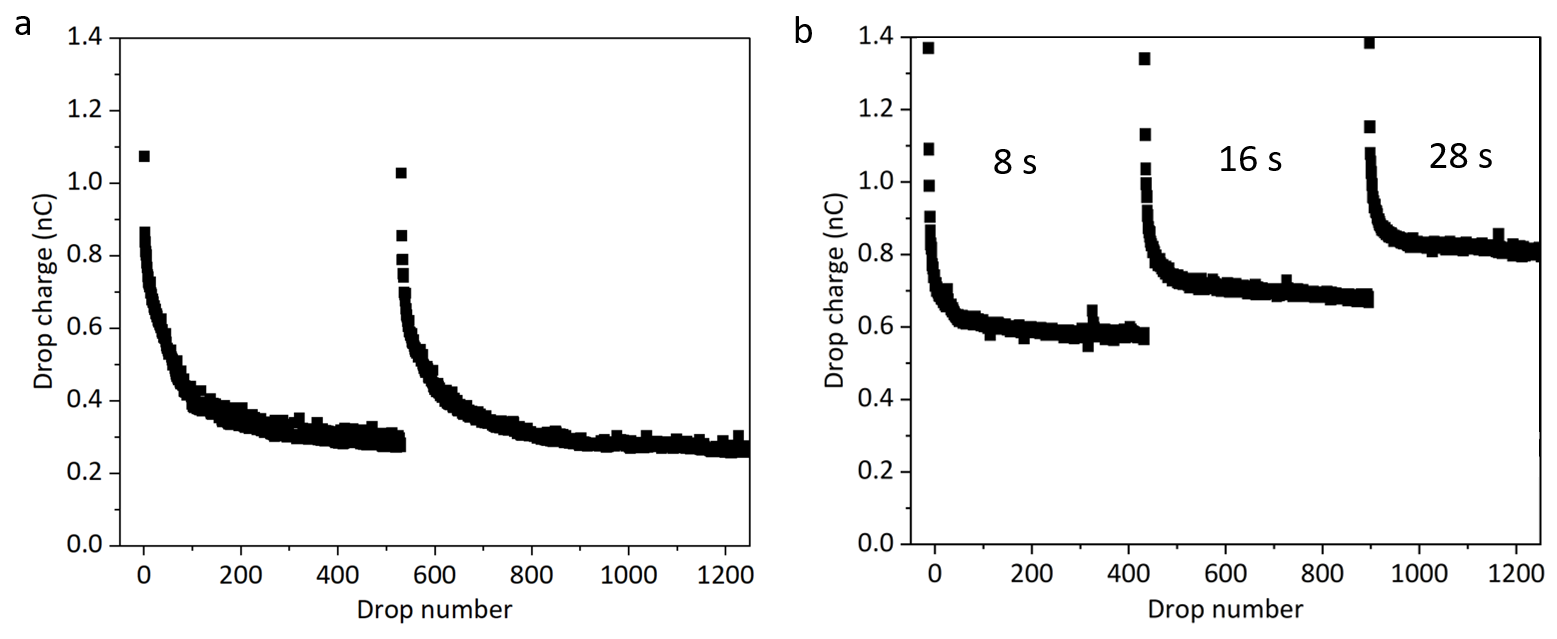


**Figure S1.** Drop charge versus drop number for 45 ± 1 µL water drops sliding over
a) PFOTS-SU8 flat surfaces at 4 s drop intervals. Then, the sliding drop was stopped intentionally after ≈500 drops, and the surface was neutralized using an ionizer air blower for 5 minutes. Afterwards, sliding drop charge was started again. The first drop after ionized air blowing had an almost similar charge as in the previous series of drops.
b) To show the independence of the neutralization phenomenon from the substrate material, the experiment (5 minutes ionizer, drops intervals: 8 s, 16 s, 28 s) was repeated, and the concept was reproduced on an inorganic substrate (glass).


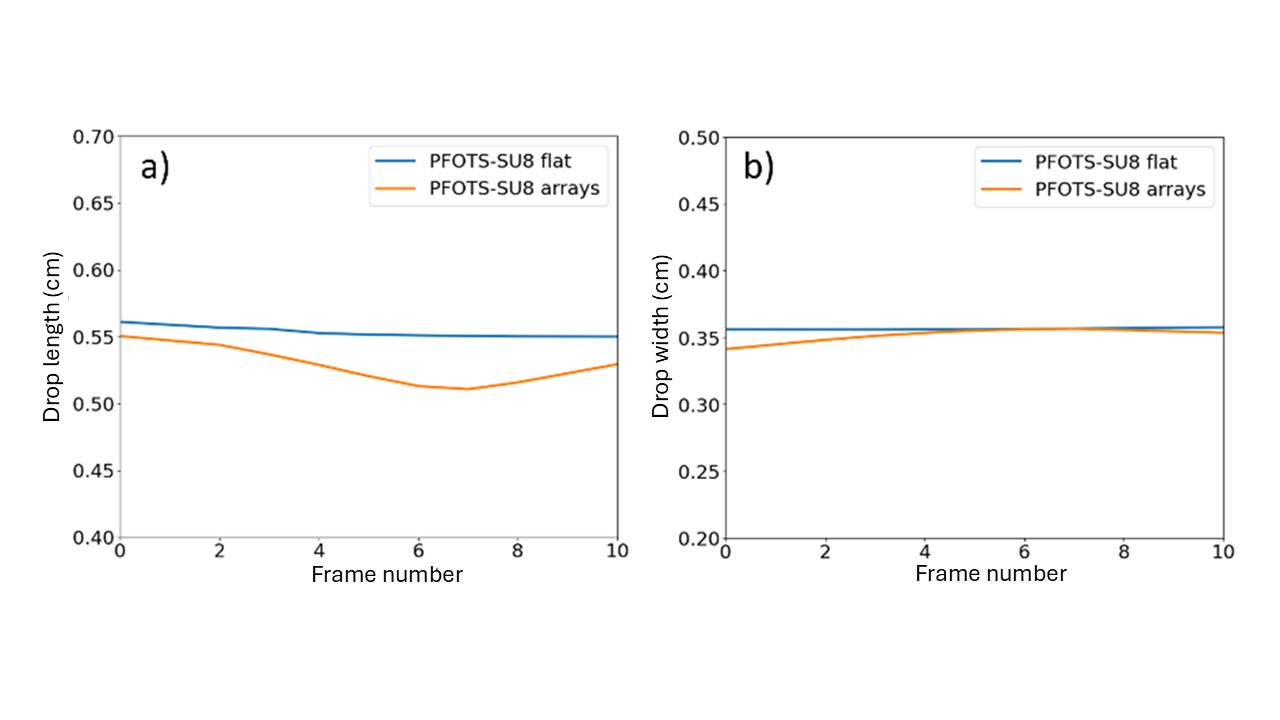


**Figure S2.** Drop geometry includes a) length and, b) width of the apparent contact area measured for PFOTS-SU8 samples sliding over 5 cm of both flat and array samples. On PFOTS-SU8 arrays, the drop length and width are consistently smaller than on the flat surface, indicating a more confined contact line. Frame number is the sequential index of the recorded frames.


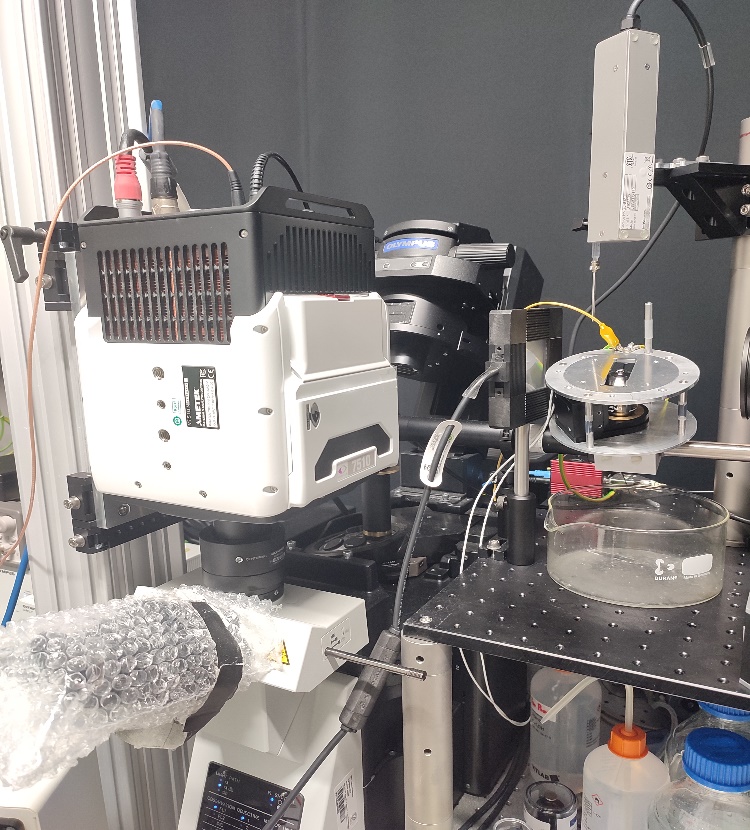


**Figure S3**. Actual image of the high-speed reflection microscope setup.


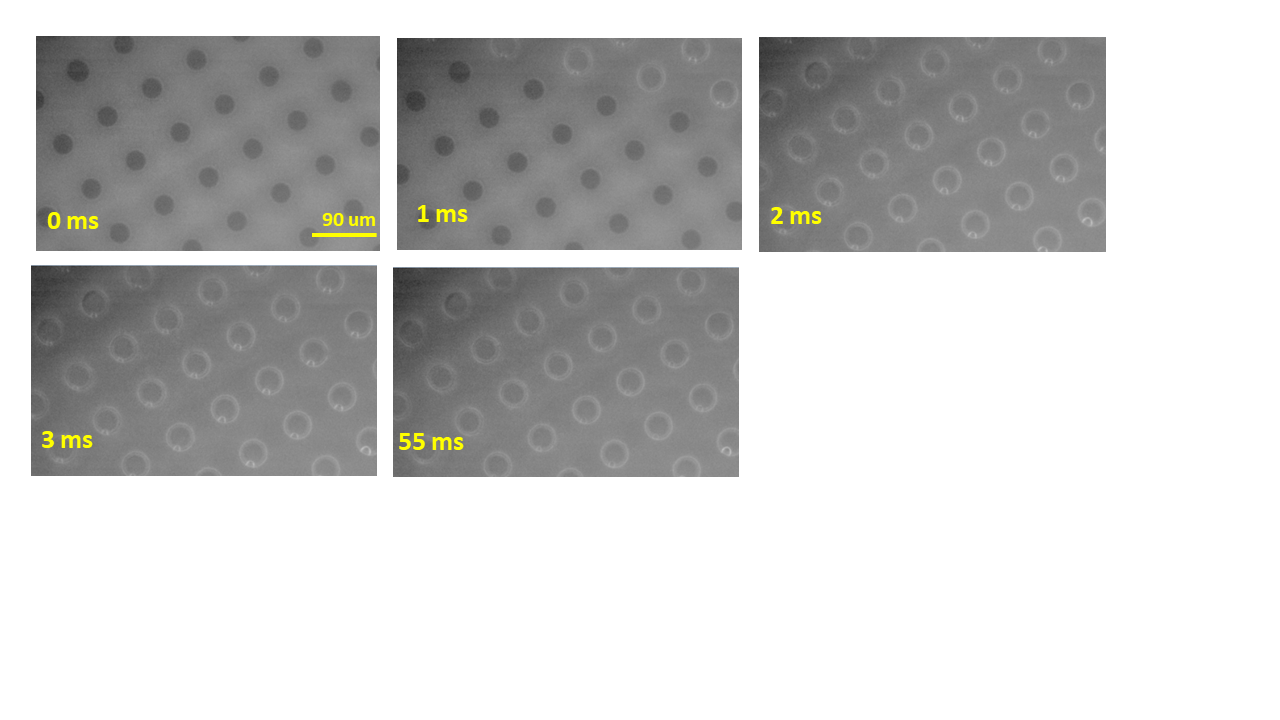


**Figure S4**. A sequence of video frames was recorded using a reflection microscope combined with a high-speed camera for the PFOTS-SU8 array at approximately 35% relative humidity (RH ≈ 35%). At 0 ms, the drop fully covered the surface. By 1 ms, the receding motion was visible in the upper-left part of the image. At 2 ms, tiny satellite droplets remained on the tips of the pillars (indicated by the red arrow). After 55 ms, the residues had fully evaporated.


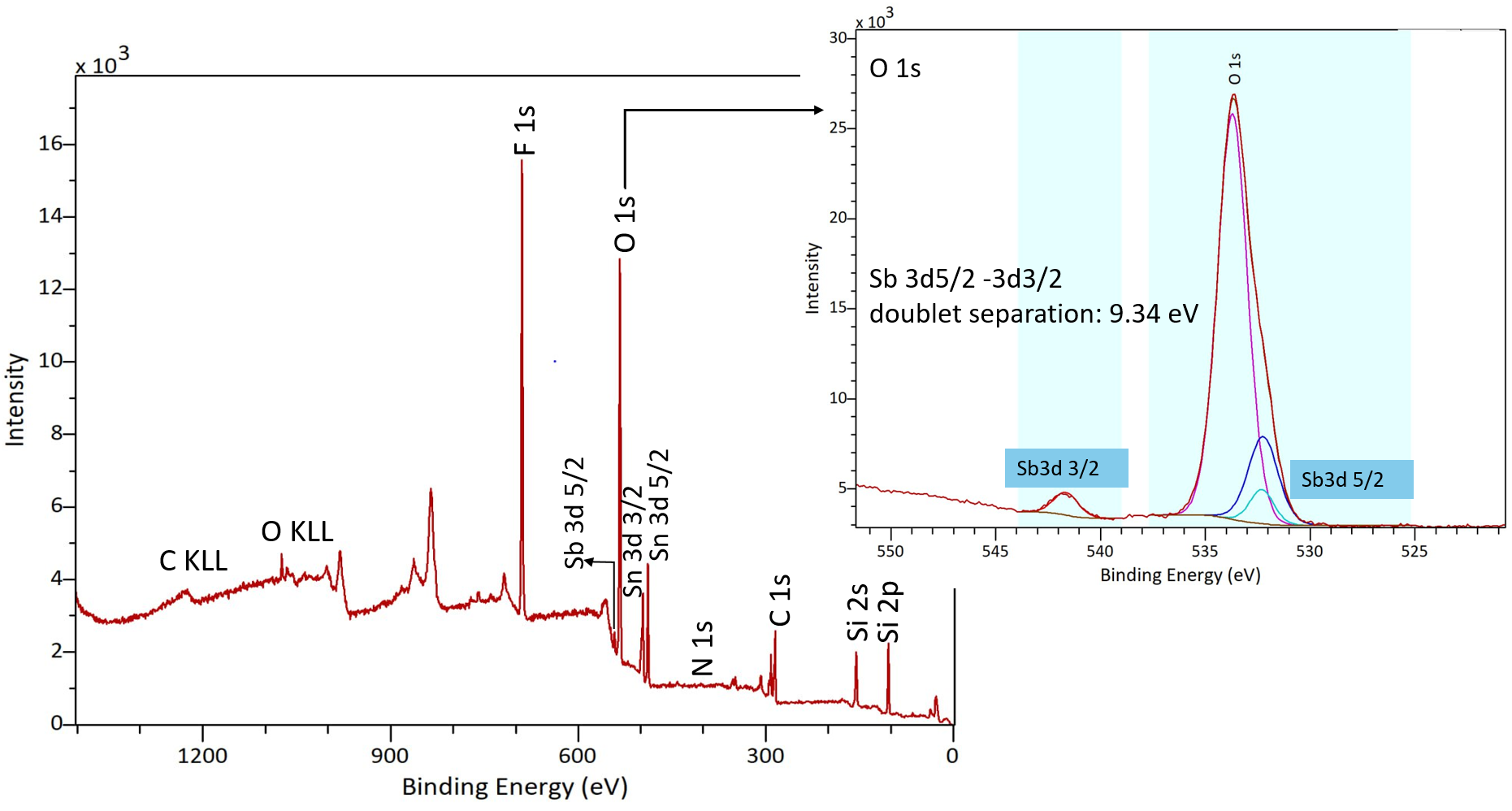


b

a

**Figure S5.** Survey scan of the N₂-SU8 Janus arrays (surface comprised of both glass and SU8 micropillar arrays). a) The XPS survey spectrum confirms both the effect of N₂ RIE plasma on PFOTS-SU8 and the effect of PFOTS on glass. Tin (Sn) doublet peaks originate from the glass substrate, as SU-8 is not expected to contain Sn. b) Antimony (Sb) is also observed. It originates from the SU-8 photoinitiator rather than from the glass substrate and overlaps with the O 1s core-level signal.


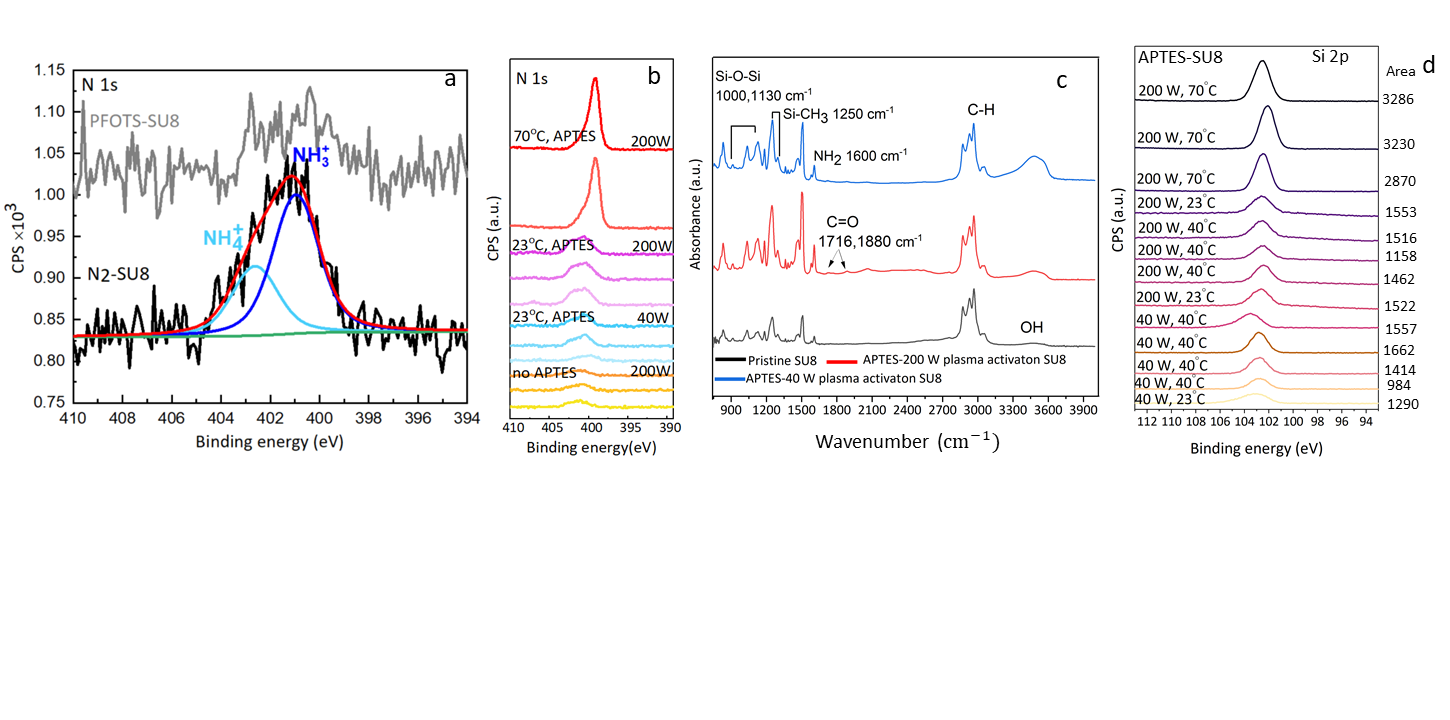


**Figure S6.** a) High-resolution scan of the N 1s region from PFOTS-SU8 before and after 200 W N₂ RIE plasma treatment. b) High-resolution scan of N 1s region of the plasma activated PFOTS-SU-8 before and after APTES deposition at varying temperatures (23 °C vs. 70 °C) and plasma powers (40 W vs. 200 W). Thermally activated APTES at 70°C intensified the N 1s signal compared to 23°C. c) Grazing Angle FTIR (GA-FTIR) spectra of APTES-SU8. The observed NH₂ peak at 1600 cm⁻¹ and Si-O-Si band between 1000-1130 cm⁻¹ confirm the possible covalent bonding of APTES. d) The peaks around 102.5 ± 0.5 eV in the Si 2p core level of APTES-SU-8 (substrate temperature during CVD: 70 °C, 23 °C, 40 °C; plasma power for activation: 200 W, 40 W) confirm the presence of siloxane, indicating covalent attachment of APTES moieties to the plasma-activated surface. The siloxane signal was significantly increased at 70 °C. Areas under each peak were calculated using curve fitting (line shape: GL(50)) in CasaXPS software.




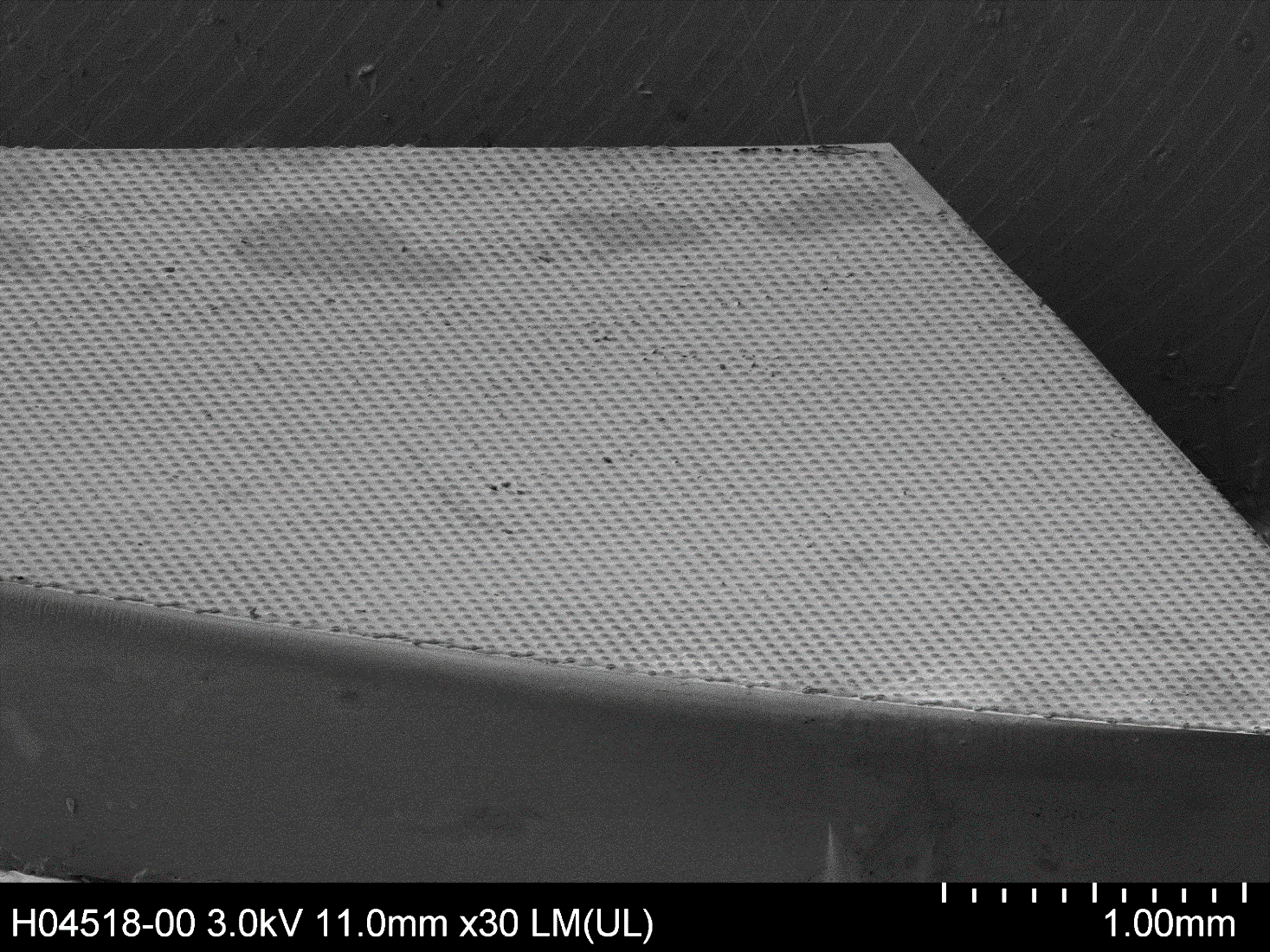

**Figure S7.** Large-scale SEM image (4 mm full width) of a fully aligned sample after development and RIE plasma treatment.

**Satellite droplet volume on the Janus sample**

1. **Spherical-cap estimate for a droplet pinned on a pillar**

A water satellite droplet deposited on top of a circular pillar is modeled as a spherical cap. The pillar diameter is d = 30 μm. The intrinsic contact angle on a flat reference sample with identical surface chemistry, measured through the liquid, is below ${10}^{\circ}$. Since such small angles cannot be determined accurately with a standard goniometer, we use $\theta={10}^{\circ}$ as a representative value of the intrinsic wetting angle. For a droplet pinned at a sharp rim, the apparent contact angle can in principle span a wide range. Apparent angles near the upper end are typically associated with advancing or forced spreading configurations, in which liquid is driven toward the pillar edge. In contrast, residues left behind by a passing primary drop form in a receding history, for which advancing-like large apparent angles are unlikely. We therefore restrict attention to moderate apparent angles and, for volume estimation, introduce an effective apparent contact angle $\theta_{\mathrm{eff}}$ in the range ${10}^{\circ}\leq\theta_{\mathrm{eff}}\leq{90}^{\circ}$. As a representative value, we use the midpoint $\theta_{\mathrm{eff}}={50}^{\circ}$ to estimate the residue volume for a given pinned footprint. To assess robustness, we additionally repeat the volume estimate for $\theta_{\mathrm{eff}}={30}^{\circ}$and ${70}^{\circ}$, which bracket moderate receding-like configurations.

The contact-line radius, $a=d/2$ is set by the pillar geometry. The droplet volume $V$ follows from the spherical-cap volume expression:

$$V=\frac{\pi h^{2}\left( 3R - h \right)}{3}. \left( 1 \right)$$

Here, $R=a/{\sin\theta_{eff}}$ is the radius of curvature of the drop and $h=R\left( 1-cos\theta_{eff} \right)$ is the height of the droplet. Using $\theta_{eff}={50}^{\circ}$ gives a radius of curvature of *R*≈19.6 μm and a height of h≈7.0 μm. Inserting these values into Eq. (1) gives

$$V\approx2.6\times{10}^{3} \mu m^{3}=2.6 pL. (2)$$

Using the same approach for $\theta_{eff}={30}^{\circ}$ and $\theta_{eff}={70}^{\circ}$, the corresponding volumes are $V\approx1.4\text{ pL}$ and $V\approx4.3\text{ pL}$, respectively.

1. **Estimate the total residue volume generated during sliding**

To determine the primary drop width $W$, we used a side-view measurement approach [10-12], which yielded $W=0.37\text{ cm}$.

To estimate the residue volume deposited on the micropillar array during sliding, the wetted contact region is approximated as a rectangle. Using a sliding distance $L=5\text{ cm}$ and adopting $W=0.37\text{ cm}$, the swept area is $A = L W = 5 \times0.37 = 1.85 cm^{2}$.

The micropillars form a square lattice with pillar diameter $d=30 \mu m$ and border-to-border spacing (gap) $g=30 \mu m$ in both directions. Thus, the center-to-center pitch was $p=d+g=60 \mu m$. The unit-cell area of the lattice is simply the pitch squared, so $A_{\mathrm{cell}}=p^{2}=(0.006\text{ }\mathrm{cm})^{2}=3.6\times{10}^{-5}\text{ }\mathrm{cm}^{2}$. Therefore, the pillar number density is the inverse of the unit-cell area, giving $n=1/A_{\mathrm{cell}}\approx2.8\times{10}^{4}$ pillars per $\mathrm{cm}^{2}$. The total number of pillars contacted within the swept area is then found by multiplying the number density by the swept area, $N=nA\approx(2.8\times{10}^{4})\times1.85\approx5.1\times{10}^{4}$ pillars.

The total residue volume is therefore

$$V_{total} = N V_{per-pillar} \approx(5.1\times{10}^{4}) \times(2.6 \times{10}^{-12}L) \approx0.13 \mu L. (3)$$

Finally, for an initial droplet volume $V_{0}=45 \mu L$, the deposited fraction after a 5 cm slide is

$$\eta=\frac{V_{total}}{V₀} \times100\% = \frac{0.13 \mu L}{45 \mu L} \times100\% \approx0.30\%\quad(4)$$

We repeated the same calculation for $\theta_{\mathrm{eff}}={30}^{\circ}$and $\theta_{\mathrm{eff}}={70}^{\circ}$. Using $V_{\text{per-pillar}}\approx1.4\text{ pL}$ and $4.3\text{ pL}$, respectively, the corresponding total deposited volumes are $V_{\text{total}}\approx0.072\text{ }\mu\text{L}$ and $V_{\text{total}}\approx0.22\text{ }\mu\text{L}$. These yields deposited fractions of $\eta\approx0.16\%$ and $\eta\approx0.49\%$, respectively. Nevertheless, $\eta$ remains well below $1\%$ across the entire plausible range.

**References:**

1. Feng, S., et al., *Tip-induced flipping of droplets on Janus pillars: From local reconfiguration to global transport.* Science advances, 2020. **6**(28): p. eabb4540.

2. Mammen, L., et al., *Functional superhydrophobic surfaces made of Janus micropillars.* Soft Matter, 2015. **11**(3): p. 506-515.

3. Chen, C.-M., C.-L. Chiang, and S. Yang, *Programming tilting angles in shape memory polymer Janus pillar arrays with unidirectional wetting against the tilting direction.* Langmuir, 2015. **31**(35): p. 9523-9526.

4. Yoon, H., et al., *Adhesion hysteresis of Janus nanopillars fabricated by nanomolding and oblique metal deposition.* Nano Today, 2009. **4**(5): p. 385-392.

5. Yoon, H., et al., *Face selection in one-step bending of Janus nanopillars.* Langmuir, 2010. **26**(12): p. 9198-9201.

6. Liu, S., et al., *Adhesion anisotropy substrate with Janus micropillar arrays guides cell polarized migration and division cycle.* Angewandte Chemie International Edition, 2019. **58**(13): p. 4308-4312.

7. Wang, T., et al., *Elliptical silicon arrays with anisotropic optical and wetting properties.* Langmuir, 2010. **26**(16): p. 13715-13721.

8. Tieqiang, W., et al., *Janus Si Micropillar Arrays with Thermal-Responsive Anisotropic Wettability for Manipulation of Microfluid Motions.* 2015.

9. Funayama, K., A. Miura, and H. Tanaka, *Flexibly designable wettability gradient for passive control of fluid motion via physical surface modification.* Scientific reports, 2023. **13**(1): p. 6440.

10. Shumaly, S., et al., *Deep Learning to Analyze Sliding Drops.* Langmuir, 2023. **39**(3): p. 1111-1122.

11. Shumaly, S., et al., *Estimating sliding drop width via side-view features using recurrent neural networks.* Scientific Reports, 2024. **14**(1): p. 12033.

12. Shumaly, S., et al. *CNN-Transformer with Absolute Positional Encoding Optimized for Low-Dimensional Inputs: Applied to Estimate Sliding Drop Width*. in *Joint European Conference on Machine Learning and Knowledge Discovery in Databases*. 2025. Springer.
